# Constituents and Anti-Multidrug Resistance Activity of *Taiwanofungus camphoratus* on Human Cervical Cancer Cells

**DOI:** 10.3390/molecules24203730

**Published:** 2019-10-16

**Authors:** Hsin-Yi Hung, Chin-Chuan Hung, Jun-Weil Liang, Chin-Fu Chen, Hung-Yi Chen, Po-Chuen Shieh, Ping-Chung Kuo, Tian-Shung Wu

**Affiliations:** 1 School of Pharmacy, College of Medicine, National Cheng Kung University, Tainan 701, Taiwan; z10308005@email.ncku.edu.tw; 2 Department of Pharmacy, College of Pharmacy, China Medical University, Taichung 402, Taiwan, hungyi@mail.cmu.edu.tw (H.-Y.C.); 3 Department of Chemistry, National Cheng Kung University, Tainan 701, Taiwan; p77625@hotmail.com; 4 Department of Life Sciences, National Cheng Kung University, Tainan 701, Taiwan; chinfu9999@gmail.com; 5 Department of Pharmacy, College of Pharmacy and Health Care, Tajen University, Pingtung 907, Taiwan; pochuen@tajen.edu.tw (P.-C.S.)

**Keywords:** *Taiwanofungus camphoratus*, chemoreversing agent, zhankuic acid, P-gp inhibitor

## Abstract

Resistance to anti-cancer drugs is one of the main factors of treatment failure resulting in high morbidity. Among the reasons of resistance, overexpression of efflux pumps leading to multidrug resistance is an important issue that needs to be solved. *Taiwanofungus camphoratus* has been used as a nutritional supplement to treat various cancers. However, its effects on the resistance to chemotherapeutic agents are still unknown. In this study, we report four new chemical constituents of *T. camphoratus* isolated from an ether extract: camphoratins K (**1**) and N (**2**) and benzocamphorins G (**3**) and I (**4**). Furthermore, we evaluated zhankuic acids A–C for their P-glycoprotein (P-gp) inhibitory effects. The results showed that zhankuic acid A was the most potent P-gp inhibitor compound and (at 20 μM) could reverse drug resistance in human cancer cells, restoring an IC_50_ of 78.5 nM for doxorubicin, of 48.5 nM for paclitaxel, and of 321.5 nM for vincristine, indicating a reversal fold of 48, 38, and 45 times, respectively. This study provides support for the use of *T. camphoratus* in the further development of cancer therapy.

## 1. Introduction

Treatment failure or metastasis are still the leading causes of death for cancer patients [1]. One of the important factors causing treatment failure is drug resistance, which can be intrinsic or acquired [2,3]. Cancer stem cells [4], tumor microenvironment, and host effects are the main reasons of intrinsic resistance, while efflux pumps, alteration of drug targets, degradation of anticancer drugs, and DNA self-repair cause acquired resistance. In particular, overexpression of efflux pumps leads to multidrug resistance (MDR) [2,3]. In order to increase the efficacy of anticancer drugs, strategies to reverse multidrug resistance are extensively studied, and P-glycoprotein (P-gp) inhibitors have evolved to the fourth generation [5,6,7,8]. However, because of their inability to of improve drug efficacy in patients as well as their toxicity, P-gp inhibitors are not yet available in the clinic. Therefore, research is now focusing on natural products, hoping to find safe and effective P-gp inhibitors [5,6,7,8].

*Taiwanofungus camphoratus*, previously named *Ganoderma camphoratum*, *Antrodia cinnamomea*, *or Antrodia camphorate* (Polyporaceae, Aphyllophorales), whose Chinese name is Zhan-Ku or Niu-Chang-Chih, is a kind of fungus parasitic to *Cinnamomum kanehirai* Hay (Lauraceae), found in the inner part of old hollow trunks. Traditionally, it has been used as a kind of medicinal food against intoxication from food, alcohol, and drugs and for its anti-diarrhea, anti-hypertensive, anti-inflammatory, and hepatoprotective effects [9]. Recent studies have revealed that Niu-Chang-Chih exerts immunomodulatory effects [10,11], anti-lung cancer effects [12,13,14], and tumor-suppressive effects in metastatic patients unresponsive to or unwilling to use chemotherapy [15]. A recent published review summarized the pharmacological effects of this mushroom [16] reporting its anticancer activity against a large variety of cancers, including breast, cervical, ovarian, prostate, bladder, colorectal, pancreatic, liver, and lung cancers, melanoma, leukemia, lymphoma, neuroblastoma, and glioblastoma. Other biological activities include anti-inflammatory, anti-atopic dermatitis, anti-cachexia, immunoregulatory, anti-obesity, anti-diabetic, anti-hyperlipidemic, anti-atherosclerotic, anti-hypertensive, anti-platelet, anti-oxidative, anti-photodamaging, hepatoprotective, renoprotective, neuroprotective, testis protecting, anti-asthmatic, osteogenic, osteoprotective, antiviral, antibacterial, and wound healing properties [16]. The major chemical constituents of this fungus are triterpenoids [17,18,19] and benzenoids [20,21]. Other components are steroids [22], diterpenoids [23], terpenoids [24], lignans [22], maleic and succinic acid derivatives [25], etc. Although the extract of *T. camphoratus* has been used as a nutritional supplement for treating cancers, the P-gp inhibitory effects of its main constituents are still unknown. Therefore, in addition to reporting new chemical constituents of *T. camphoratus*, this study reveals the P-gp inhibitory effects of zhankuic acids A–C.

## 2. Results and Discussion

### 2.1. Purification and Identification of Chemical Constituents

The basswood cultivated fruiting bodies of *T. camphoratus* (3.6 kg) were repeatedly extracted with ether (4 × 10 L) for 3 days. The ether extract was concentrated in vacuo to afford a brown syrup (370 g) and then partitioned between water and ether. The ether layer was chromatographed repeatedly over silica gel, as described in Supplementary Materials. In total, 45 compounds were obtained. Among them, two triterpenoids, camphoratins K (**1**) and N (**2**), and two benzenoids, benzocamphorins G (**3**) and I (**4**), were isolated and characterized from *T. camphoratus* for the first time. Other known isolated compounds were triterpenoids, including methyl antcinate A (**5**), antcins A (**6**), C (**12**), and K (**18**), zhankuic acid A methyl ester (**7**), zhankuic acids A (**8**), B (**11**), C (**9**), and D (**10**), camphoratins E (**13**) and F (**14**), methyl antcinate (**15**), antcamphins A (**19**), B (**16**), and D (**17**); terpenoids, including 1-hydroxy-*p*-menth-3-en-2-one (**20**), nerolidol (**21**), coenzyme Q (**22**), 4-acetylantroquinonol B (**23**); steroids, including ergosterol (**24**), ergosterol peroxide (**25**), camphoratin I (**26**); lignans, including sesamin (**27**) and 4-hydroxysesamin (**28**); and benzenoids, including antrocamphins A (**30**) and B (**29**), benzocamphorins C (**37**), D (**44**), E (**43**), F (**31**), and H (**32**), methyl 3,4,5-trimethoxybenzoate (**33**), methyl 2,3,4,5-tetramethoxy benzoate (**34**), 1-methyl-2,3,4,5-trimethoxy benzene (**35**), 2,3,6-trimethoxy-5-methylphenol (**36**), methyl 2,5-dimethoxy-3,4-methylenedioxybenzoate (**38**), 4,5-dimethoxy-6-methyl-1,3-benzodioxole (**39**), 4,7-dimethoxyl-5-methyl-1,3-benzodioxole (**40**), 2,3-(methylenedioxy)-4-methyl-5-methylphenol (**41**), 2,2,5,5-tetramethoxyl-3,4,3,4-bimethylenedioxyl-6,6-dimethylbiphenyl (**42**), and tetracanyl ferulate (**45**) (see Supplementary Materials for their references).

### 2.2. Structural Elucidations of Camphoratins K (**1**) and N **(2**) and Benzocamphorins G (**3**) and I (**4**)

**Figure 1:**
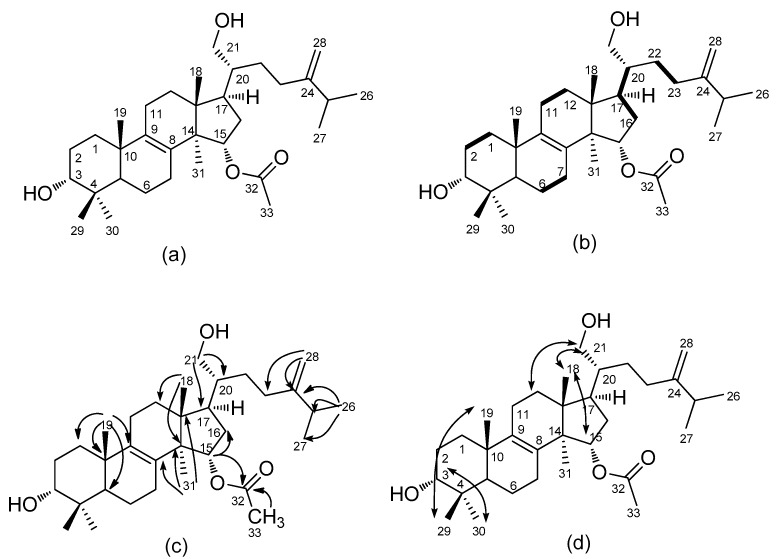
Structure of camphoratin K (**1**) (**a**) and its key COSY (**b**), HMBC (**c**), and NOESY (**d**) correlations.

Camphoratin K (**1**) was isolated as a white powder, and its sodiated molecular formula, C_33_H_54_O_4_Na, was established from a sodium adduct ion peak at *m*/*z* 537.3917 in high-resolution electrospray ionization mass spectrometric (HR-ESI–MS) analysis. The infrared (IR) absorption bands at 3427, 1714, 1643, 1455, and 891 cm^−1^ were in agreement with the presence of a hydroxyl group, an ester, and a terminal double bond. In its ^1^H-NMR spectrum, there were proton signals for five methyl singlets at δ 0.80 (6H, s, CH_3_-18, 29), 0.97 (3H, s, CH_3_-19), 0.99 (3H, s, CH_3_-30), 1.01 (3H, s, CH_3_-31), 2.04 (3H, s, CH_3_-33), two methyl doublets at δ 1.01 (3H, d, *J* = 6.8 Hz, CH_3_-27) and 1.02 (3H, d, *J* = 6.8 Hz, CH_3_-26), and four protons at δ 2.22 (1H, sept, *J* = 6.8 Hz, H-25), 3.22 (1H, dd, *J* = 4.4 Hz, 11.6 Hz, H-3), 3.69 (2H, m, H-21), 5.05 (1H, dd, *J* = 5.8, 9.4 Hz, H-15). The ^13^C-, DEPT- and HMQC NMR spectra showed 33 carbon signals composed of 8 methyls at δ 15.4, 16.4, 18.3, 19.1, 21.4, 21.8, 21.9, 28.0; 9 methylenes at δ 18.2, 20.8, 26.4, 27.7, 28.2, 30.6, 31.4, 35.5, 36.0; 1 oxygenated methylene at δ 62.0; 2 oxygenated methines at δ 76.0, 78.9, and 1 terminal olefinic carbon at δ 106.4, which indicated a triterpene skeleton. An acetyl group was assigned to link to C-15 from HMBC spectral correlations of H-15 (δ 5.05) to C-16 (δ 36.0) and C-32 (δ 171.1). A terminal olefinic group and an isopropyl group were built up via ^2^*J*, ^3^*J*-HMBC correlations of CH_3_-26 (δ 1.02) to C-24 (δ 156.1), C-25 (δ 33.7), and C-27 (δ 21.9) and H-28 (δ 4.73 and 4.67) to C-23 (δ 31.4), C-24 (δ 156.1), and C-25 (δ 33.7). Other HMBC correlations indicated a sulphurenic acid skeleton with a hydroxyl methylene [H-21 (δ 3.69) to C-17 (δ28.0)/C-20 (δ 43.2)] instead of an acid connected to C-20. This C-21 hydroxyl substitution is novel and rare among all isolates from *T.* *camphoratus* (Figure 1).

**Figure 2:**
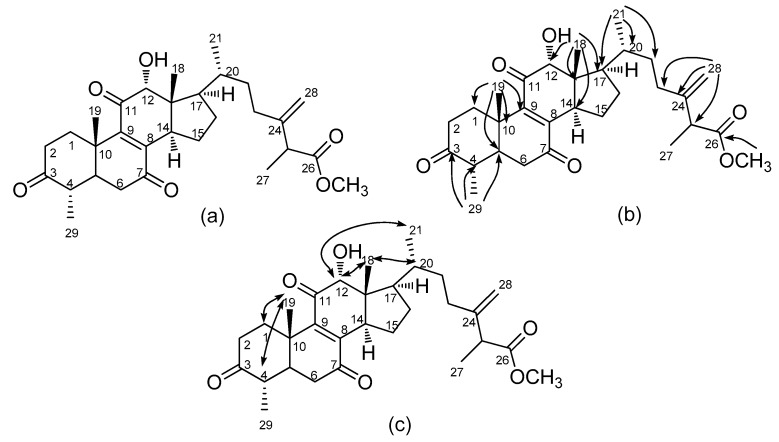
Structure of camphoratin N (**2**) (**a**) and its key HMBC (**b**) and NOESY (**c**) correlations.

Camphoratin N (**2**) appeared as a pale yellow solid with sodiated molecular formula C_30_H_42_O_6_Na (*m*/*z* 521.2876). The presence of an 8(9)-ene-7,11-dione moiety was proposed along with those of a carboxyl group and a hydroxyl group, according to a UV maximum at 267 nm and IR absorptions at 3495, 1736, 1713, 1674, 1458, and 901 cm^−1^, respectively. Comparison of its ^13^C-NMR data with those of antcamphin I [18] indicated the presence of an additional oxygenated methyl group. Assignment of the oxygenated methyl group attached to a terminal carboxylate was based on the HMBC correlation of CH_3_-26 (δ 3.67) to C-26 (δ 175.0). Also, 12α-OH and 29α-CH_3_ were suggested via the NOE enhancements of H-12 (δ 4.11)/CH_3_-18 (δ 0.67) and CH_3_-19 (δ 1.54)/H-4 (δ 2.43) (Figure 2). According to a previous study, camphoratin N could include a pair of epimers (25 *S/R*) that have identical NMR data [18].

**Figure 3:**
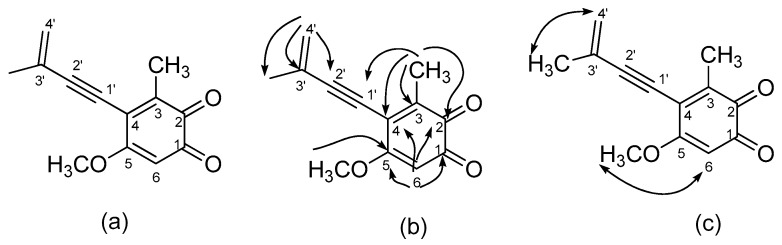
Structure of benzocamphorin G (**3**) (**a**) and its key HMBC (**b**) and NOESY (**c**) correlations.

Benzocamphorin G (**3**), a colorless syrup, was isolated via thin-layer chromatography and has the pseudomolecular formula of C_13_H_12_O_3_Na, constructed from the sodiated peak at *m*/*z* 239.0686 in HR-ESI–MS analysis. Characteristic absorption bands in its IR spectrum revealed alkynes (2205 cm^−1^), conjugated carbonyls (1667 cm^−1^), and alkenes (1607 cm^−1^). UV absorption maxima were at 268 and 296 nm. A comparison of its NMR spectra data with those of antrocamphin A [18] indicated similar proton peaks at δ 5.44 (1H, s, terminal alkene), 5.55 (1H, s, terminal alkene), 3.82 (3H, s, OCH_3_), 2.22 (3H, s, CH_3_-3), and 2.01 (3H, s, CH_3_-3′) as well as downfield shift of one aromatic proton (δ 5.99, 1H, s, H-6) and loss of two methoxy signals. ^13^C- and DEPT-135 NMR spectra revealed a similar pattern to that of antrocamphin A, except for a pair of *ortho*-carbonyls (δ 181.1 and 181.3), a downfield shift of C-6 (δ 107.0), and a loss of two methyl carbons. The positions of the *ortho*-carbonyls were assigned to be at C-1 and C-2 via HMBC correlations of H-6 (δ 5.90)/ with C-1 (δ 183.1), with C-2 (δ 181.3), with C-4 (δ 129.4), with C-5 (δ 158.8); and of CH_3_-3 (δ 2.22) with C-2 (δ 181.3), with C-3 (δ 144.6), with C-4 (δ 129.4), with C-1′ (δ 81.7), respectively. Therefore, the structure of benzocamphorin G was established and is shown in Figure 3.

**Figure 4:**
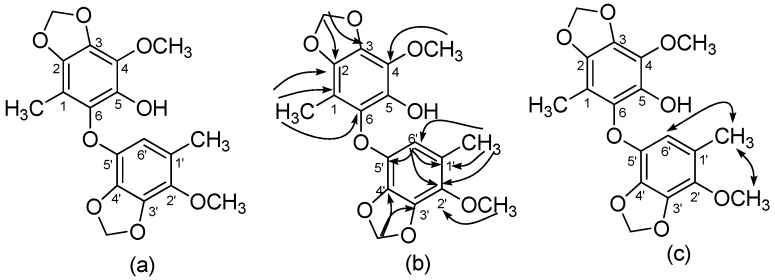
Structure of benzocamphorin I (**4**) (**a**) and its key HMBC (**b**) and NOESY (**c**) correlations.

Benzocamphorin I (**4**) was also isolated via thin-layer chromatography as colorless syrup. HR-ESI–MS analysis indicated its pseudomolecular formula as C_18_H_18_O_8_Na (*m*/*z* 385.0898). No alkynes and alkene characteristic peaks were detected in its IR spectrum. Three pairs of proton signals revealed two methyls (δ 1.99, 2.05), two methylenedioxy groups (δ 5.98, 6.00), and two methoxys (δ 3.89, 3.90). Further ^13^C-, DEPT, and HSQC spectra indicated a benzocamphorin D skeleton [19] with an oxygen linkage between two phenyl groups. However, one methoxy signal was lacking, and an oxygenated aromatic carbon was present compared to benzocamphorin D [19], showing the methoxy group was replaced by a hydroxyl group. Thus, the structure of benzocamphorin I was determined and is shown in Figure 4.

### 2.3. P-gp Inhibitory Effects of the Extract of T. camphoratus

**Figure 5:**
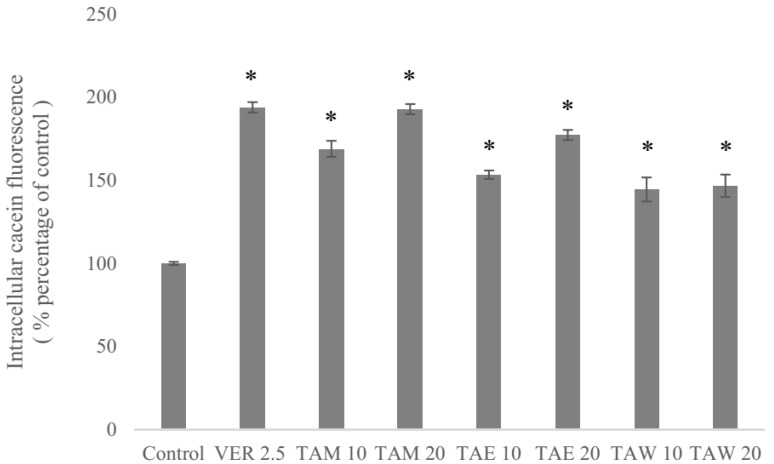
The inhibitory effects of the methanol extract at 10 and 20 μM concentrations (TAM 10, TAM 20), the EtOAc layer (TAE 10, TAE 20), the water layer (TAW 10, TAW 20) and verapamil at 2.5 μM concentration (VER 2.5) on P-glycoprotein (P-gp) in *ABCB1*/Flp-In^TM^-293 cells. * denotes *p* < 0.05 compared with the intracellular calcein fluorescence in the control group. The numbers, 2.5, 10, 20, indicate the μM concentrations.

A pilot study using methanol as a solvent was done to evaluate the P-gp inhibitory effects of the extract of *T. camphoratus* (Figure 5). The methanol extract was further partitioned using water and EtOAc. Therefore, the methanol extract (TAM), the EtOAc layer (TAE), and the water layer (TAW) were tested using human stably P-gp-expressing cells (ABCB1/Flp-InTM-293) in a calcein AM (acetoxymethyl) uptake assay [26]. The increased intracellular calcein fluorescence corresponded to the inhibition level of P-gp efflux function. The methanol extract as well as the EtOAc layer and the water layer exhibited P-gp inhibitory activities at concentrations of 10 and 20 μM. The methanol extract and the EtOAc layer exhibited inhibition in a dose-dependent manner. Moreover, the methanol extract at 20 μM (TAM 20) showed P-gp inhibition comparable to that of the first-generation P-gp inhibitor verapamil at a concentration of 2.5 μM.

### 2.4. Zhankuic Acids A–C Inhibited P-gp Efflux Function

**Figure 6:**
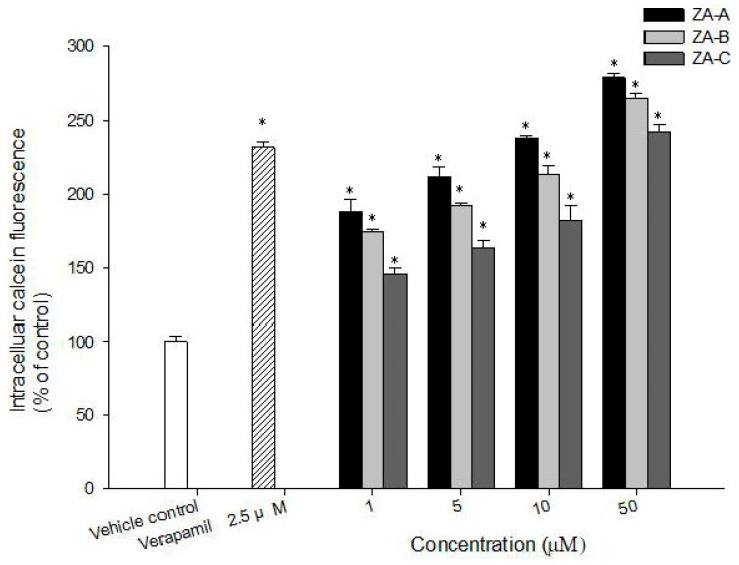
The concentration-dependent inhibitory effects of zhankuic acids (ZAs) A, B, C on P-gp in *ABCB1*/Flp-In^TM^-293 cells. * denotes *p* < 0.05 compared with the intracellular calcein fluorescence in control group.

Although four new compounds were isolated, they were in little amount. In order to better understand the main P-gp inhibitory effects of *T. camphoratus*, three of its major components, zhankuic acids (ZAs) A–C, were evaluated for their ability to inhibit P-gp using the calcein AM uptake assay. ZAs A, B, C inhibited P-gp efflux function in a concentration-dependent manner (Figure 6). Among the tested compounds, ZA-A demonstrated the most significant P-gp inhibitory effect.

### 2.5. The MDR Reversal Effects of ZAs A, B, C

To examine the MDR reversal effects of ZAs A, B, C, the cytotoxicity of a combination of these triterpenoids and chemotherapeutic drugs was evaluated in HeLaS3 and MDR KBvin cells. The IC_50_ of doxorubicin, paclitaxel, and vincristine in HeLa cells were 104 nM, 4.65 nM, and 41.5 nM, while in KBvin cells they were 3750 nM, 1824 nM, and 14,540 nM, indicating high multidrug resistance of the cells. When ZAs A–C were combined with the chemotherapeutic agents, the IC_50_ of doxorubicin, paclitaxel, and vincristine in MDR KBvin cells were significantly decreased (Table 1). Reversal folds were calculated by dividing the IC_50_ of the individual chemotherapeutic drug by the IC_50_ of the compound–drug combinations. ZA-A possessed the most significant MDR reversal effect among the tested compounds. It (20 μM) reversed drug resistance leading to an IC_50_ of 78.5 nM for doxorubicin, of 48.5 nM for paclitaxel, and of 321.5 nM for vincristine, corresponding to reversal folds of 48, 38, and 45, respectively.

## 3. Materials and Methods

### 3.1. General

The spectroscopic data of the purified compounds including optical rotations (${\left[\alpha \right]}_{D}^{25}$), UV, and IR spectra were recorded on a Jasco P-2000 digital polarimeter (Jasco, Tokyo, Japan), a Hitachi U-0080D diode array spectrophotometer (Hitachi, Tokyo, Japan), and a Jasco FT/IR-4100 spectrophotometer (Jasco, Tokyo, Japan), respectively. The mass spectra were collected on a Shimadzu LC-MS 8040 spectrometer (Shimadzu, Kyoto, Japan). The HRMS data were obtained on a JMS-T100LP spectrometer (Jeol, Tokyo, Japan). ^1^H-, ^13^C-, and 2D NMR spectra were recorded on the Bruker AV-500 and Avance III-400 NMR spectrometers (Bruker, Billerica, MA, USA). The deuterated solvents were purchased from Sigma-Aldrich (St. Louis, MO, USA). Other chemicals used in this study were provided by Merck KGaA (Darmstadt, Germany). Column chromatography was performed on silica gels in different mesh sizes (70–230 and 230–400 mesh, Kieselgel 60, Merck KGaA, Darmstadt, Germany). Thin-layer chromatography (TLC) was conducted on precoated Kieselgel 60 F 254 plates (Merck KGaA, Darmstadt, Germany). The spots on TLC were detected by UV light or spraying with 10% (*v*/*v*) H_2_SO_4_ followed by heating at 110 °C for 10 min.

### 3.2. Plant Materials

The fresh fruiting bodies of *T. camphoratus* were provided by TWHERB Biomedical Co., LTD, Hsinchu, Taiwan (APACC-OG-100-034) in September 2009. The fungus was identified by Dr. Tun-Tschu Chang (Taiwan Forestry Research Institute, Taipei, Taiwan). A voucher specimen (TSWu 2009-001-010) was deposited in the School of Pharmacy, National Cheng Kung University, Tainan, Taiwan.

### 3.3. Extraction and Isolation

The fruiting bodies of *T. camphoratus* (3.6 kg) were extracted with Et_2_O (4 × 10 L) for 3 days. The Et_2_O extract was concentrated to afford a brown syrup (370 g) and then partitioned between H_2_O and Et_2_O. The ether layer was chromatographed on silica gel and eluted with MeOH in chloroform (0–100% of MeOH, gradient) to obtain 10 fractions, (Fractions 1–10) monitored by TLC. Fraction 2 underwent silica gel column chromatography using *n*-hexane–EtOAc (10:1) to obtain compound **3** (7 mg). Fractions 3 and 4 were combined and subjected to silica gel column chromatography, eluted successively with a step gradient of *n*-hexane–EtOAc (3:1 to 1:2) to yield compounds **1** (15 mg) and **4** (7 mg). Fractions 5 and 9 were combined and chromatographed on a column of silica gel, eluted successively with a step gradient of CHCl_3_–MeOH as eluent to yield compound **2**.

#### 3.3.1. Camphoratin K (**1**)

Colorless powder; ${\left[\alpha \right]}_{D}^{25}$ +111.2 (c 0.2, MeOH); IR (KBr) ν_max_: 3427, 2959, 2942, 2885, 1714, 1643, 1455, 1374, 1266, 1249, 1031, 891 cm^−1^; UV (MeOH) λ_max_: 243, 253 nm; ESI–MS *m*/*z* 537 [M + Na]^+^; HR-ESI-MS *m*/*z* 537.3917 ([M + Na]^+^) (Calcd. for C_33_H_44_O_4_Na: 531.3920); ^1^H-NMR (CDCl_3_, 400 MHz) *δ* 5.05 (1H, dd, *J* = 5.8, 9.4 Hz, H-15), 4.73 (1H, s, H-28), 4.67 (1H, s, H-28), 3.67 (3H, s, OCH_3_), 3.69 (1H, m, H-21), 3.22 (1H, dd, *J* = 4.4, 11.6 Hz, H-3), 2.22 (1H, sept, *J* = 6.8 Hz, H-25), 2.12 (1H, m, H-16), 2.11 (1H, m, H-23), 2.10 (1H, m, H-7), 2.04 (1H, m, H-11), 2.03 (1H, m, H-7), 2.01 (1H, m, H-20), 1.93 (1H, m, H-12), 1.91 (2H, m, H-11, H-23), 1.73 (1H, m, H-1), 1.63 (2H, m, H-2, H-22), 1.61 (1H, m, H-12), 1.56 (1H, m, H-6), 1.55 (1H, m, H-2), 1.50 (1H, m, H-22), 1.48 (1H, m, H-6), 1.46 (1H, m, H-17), 1.22 (1H, m, H-1), 1.02 (3H, d, *J* = 6.8 Hz, H-26), 1.02 (3H, s, H-31), 1.01 (1H, m, H-5), 1.01 (3H, d, *J* = 6.8 Hz, H-27), 0.99 (3H, s, H-30), 0.97 (3H, s, H-19), 0.80 (3H, s, H-18), 0.80 (3H, s, H-29). ^13^C-NMR (CDCl_3_, 100 MHz) *δ* 171.1 (C-32), 156.1 (C-24), 135.5 (C-9), 132.8 (C-8), 106.4 (C-28), 78.9 (C-3), 76.0 (C-15), 62.0 (C-21), 51.0 (C-13), 50.1 (C-5), 44.4 (C-14), 43.2 (C-20), 38.8 (C-4), 37.1 (C-10), 36.0 (C-16), 35.5 (C-1), 33.7 (C-25), 31.4 (C-23), 30.6 (C-12), 28.2 (C-6), 28.0 (C-17), 28.0 (C-31), 27.7 (C-2), 26.4 (C-11), 21.9 (C-27), 21.8 (C-26), 21.4 (C-33), 20.8 (C-7), 19.1 (C-19), 18.3 (C-31), 18.2 (C-22), 16.4 (C-18), 15.4 (C-29).

#### 3.3.2. Camphoratin N (**2**)

Yellow solid; ${\left[\alpha \right]}_{D}^{25}$ +194.9 (c 0.1, MeOH); IR (KBr) ν_max_: 3495, 2944, 2926, 2892, 1736, 1713, 1674, 1458, 1378, 1238, 1200, 1167, 1065, 901 cm^−1^; UV (MeOH) λ_max_: 267 nm; ESI–MS *m*/*z* 521 [M + Na]^+^; HR-ESI-MS *m*/*z* 521.2876 ([M + Na]^+^) (Calcd. for C_30_H_42_O_6_Na: 521.2879); ^1^H-NMR (CDCl_3_, 400 MHz) *δ* 4.91 (1H, s, H-28), 4.87 (1H, s, H-28), 4.11 (1H, s, H-12), 3.67 (3H, s, OCH_3_), 3.13 (1H, q, *J* = 7.1 Hz, H-25), 3.03 (1H, dd, *J* = 7.3, 12.4 Hz, H-14), 2.93 (1H, ddd, *J* = 2.7, 7.0, 2.7 Hz, H-1), 2.55 (1H, m, H-6), 2.54 (1H, m, H-2), 2.45 (1H, m, H-6), 2.43 (1H, m, H-4), 2.41 (1H, m, H-2), 2.40 (1H, m, H-15), 2.11 (1H, m, H-23), 1.99 (1H, m, H-16), 1.99 (1H, m, H-5), 1.96 (1H, m, H-23), 1.87 (1H, m, H-17), 1.58 (1H, m, H-22), 1.55 (1H, m, H-16), 1.54 (3H, s, H-19), 1.47 (1H, m, H-1), 1.44 (1H, m, H-15), 1.42 (1H, m, H-20), 1.28 (3H, d, *J* = 7.1 Hz, H-27), 1.20 (1H, m, H-22), 1.04 (3H, d, *J* = 6.6 Hz, H-29), 0.97 (3H, d, *J* = 6.5, H-21), 0.67 (3H, s, H-18). ^13^C-NMR (CDCl_3_, 100 MHz) *δ* 210.7 (C-3), 201.9 (C-11), 200.1 (C-7), 150.3 (C-9), 148.4 (C-24), 145.3 (C-8), 80.4 (C-12), 49.3 (C-13), 48.5 (C-5), 45.6 (C-17), 43.9 (C-4), 41.7 (C-14), 39.0 (C-6), 37.9 (C-10), 37.5 (C-2), 35.3 (C-20), 34.5 (C-1), 33.8 (C-22), 31.2 (C-23), 26.8 (C-16), 23.8 (C-15), 17.9 (C-21), 16.3 (C-19), 11.4 (C-18).

#### 3.3.3. Benzocamphorin G (**3**)

Colorless syrup; IR (KBr) ν_max_: 2937, 2205, 1667, 1607, 1588, 1450, 1365, 1273, 1230, 1078, 853 cm^−1^; UV (MeOH) λ_max_: 268, 296 nm; ESI-MS *m*/*z* 239 [M + Na]^+^; HR-ESI–MS *m*/*z* 239.0686 ([M + Na]^+^) (Calcd. for C_13_H_12_O_3_Na: 239.0684); ^1^H-NMR (CDCl_3_, 400 MHz) *δ* 5.90 (1H, s, H-2), 5.55 (1H, s, H-4′), 5.44 (1H, s, H-4), 3.82 (3H, s, OCH_3_), 2.22 (3H, s, CH_3_-5), 2.01 (3H, s, CH_3_-3′). ^13^C-NMR (CDCl_3_, 100 MHz) *δ* 183.1 (C-1), 181.3 (C-6), 158.8 (C-3), 144.6 (C-5), 129.4 (C-4), 126.2 (C-3′), 125.1 (C-4′), 108.7 (C-2′), 107.0 (C-2), 81.7 (C-1′), 56.3 (OCH_3_), 23.0 (CH_3_-3′), 14.5 (CH_3_-5).

#### 3.3.4. Benzocamphorin I (**4**)

Colorless syrup; IR (KBr) ν_max_: 3276, 2936, 2892, 1496, 1457, 1443, 1232, 1115, 1070, 1051, 956 cm^−1^; UV (MeOH) λ_max_: 265, 284 nm; ESI-MS *m*/*z* 385 [M + Na]^+^; HR-ESI–MS *m*/*z* 385.0898 ([M + Na]^+^) (Calcd. for C_18_H_18_O_8_Na: 385.0899); ^1^H-NMR (CDCl_3_, 400 MHz) *δ* 6.00 (2H, OCH_2_O-2′,3′), 5.98 (2H, OCH_2_O-3′,4′), 5.97 (1H, s, H-6′), 5.17 (1H, s, OH-5), 3.90 (3H, s, OCH_3_-4), 3.89 (3H, s, OCH_3_-2′), 2.05 (3H, s, CH_3_-1′), 1.99 (3H, s, CH_3_-1). ^13^C-NMR (CDCl_3_, 100 MHz) *δ* 136.8 (C-3, 137.4 (C-2′), 136.1 (C-6, C-3′), 135.5 (C-5′), 135.0 (C-4), 134.4 (C-2), 133.4 (C-4′), 129.4 (C-5), 116.9 (C-1), 109.5 (C-6′), 101.8 (OCH_2_O-3′,4′), 101.8 (OCH_2_O-2, 3), 60.1 (OCH_3_-4), 60.0 (OCH_3_-2), 59.8 (OCH_3_-2′), 15.9 (CH_3_-1′), 9.4 (CH_3_-1).

### 3.4. Culture of Cell Lines

Human stably P-gp-expressing cells (ABCB1/Flp-InTM-293) were established and cultured in DMEM as in a previous study [27]. The human cervical epithelioid carcinoma cell line HeLaS3 was purchased from Bioresource Collection and Research Center (Hsinchu, Taiwan), and the multi-drug resistant human cervical cancer cell line KBvin was kindly provided by Dr. Kuo-Hsiung Lee (University of North Carolina, Chapel Hill, NC, USA). All cancer cell lines were cultured in RPMI-1640 containing 10% FBS, at 37 °C in a humidified atmosphere of 5% CO_2_.

### 3.5. Calcein AM Uptake Assay

The calcein AM uptake assay was performed to evaluate the inhibitory effect of the test compounds on human P-gp efflux function. To be brief, 1 × 10^5^ cells/well were seeded in 96-well black plates overnight. Before starting the assay, the cells were washed and pre-incubated with warm Hanks′ balanced salt solution (HBSS) for 30 min. Then, the test compounds were added, and incubation was carried out for 30 min. Calcein-AM was added after washing with warm PBS. The BioTek Synergy HT Multi-Mode Microplate Reader was utilized to detect calcein fluorescence (excitation/emission wavelength = 485 nm/528 nm) at 37 °C every 3 min for 30 min. Each experiment was performed at least three times, each in triplicate on different days.

### 3.6. SRB Cytotoxicity Assay and Reversal Fold Calculation

Briefly, after 72 h of treatment with series concentrations of chemotherapeutic drugs with or without the test compounds, 50% trichloroacetic acid (TCA) was added to fix the cells for 30 min. After air-drying, the cells were stained with 0.04% sulforhodamine B (SRB) for 30 min and washed with 1% acetic acid. The bound stain was solubilized in 10 mM Tris base, and the absorbance was measured by a Synergy HT Multi-Mode Microplate Reader (BioTek, Winooski, VT, USA) at 515 nm. Reversal folds were calculated by dividing the IC_50_ of each drug by the IC_50_ of the compound–drug combination treatment.

## 4. Conclusions

Although numerous anti-cancer drugs are marketed, resistance to cancer treatments is still the top reason for cancer death. Multidrug resistance is largely due to the high expression of efflux pumps. *T. camphoratus*, a medicinal fungus, was reported to exhibit anti-cancer properties, but its effects toward cancer multidrug resistance are unknown. In this study, four new chemical constituents, camphoratins K (**1**) and N (**2**) and benzocamphorins G (**3**) and I (**4**), were reported for the first time, and the main constituents of *T. camphoratus*, zhankuic acids A–C, were found to have P-gp inhibitory effects in a dose dependent manner. In addition, zhankuic acid A (20 μM), the most potent P-gp inhibitor, could effectively reverse MDR in KBvin cells, leading to an IC_50_ of 78.5 nM for doxorubicin, of 48.5 nM for paclitaxel, and of 321.5 nM for vincristine, corresponding to reversal folds of 48, 38, and 45, respectively.

## Figures and Tables

**Table 1:** The cytotoxic IC_50_ and reversal fold of drug resistance for ZAs A, B, C in combination with chemotherapeutic drugs in HeLaS3 and MDR KBvin cells.

	HeLa		KBvin	
	IC_50_ ± SD (nM)	RF ^1^	IC_50_ ± SD (nM)	RF ^1^
Doxorubicin	104.5 ± 6.36	1	3750 ± 70.7	1
+Verapamil (2.5 μM)	83.61 ± 3.12 *	1.2	705.21 ± 19.13 *	5.3
+ZA-A (10 μM)	76.000 ± 1.41 *	1.4	420 ± 56.6 *	8.9
+ZA-A (20 μM)	51.500 ± 2.12 *	2	78.5 ± 3.53 *	47.8
+ZA-B (10 μM)	103.000 ± 1.43	1	2050 ± 72.5	1.8
+ZA-B (20 μM)	66.500 ± 4.94 *	1.6	1200 ± 23.5 *	3.1
+ZA-C (10 μM)	101.500 ± 2.12	1	2100 ± 25.3	1.8
+ZA-C (20 μM)	83.000 ± 1.42 *	1.3	1800 ± 45.7 *	2.1
Paclitaxel	4.65 ± 0.21	1	1824 ± 125.87	1
+Verapamil (2.5 μM)	0.95 ± 0.03 *	4.9	75.81 ± 4.95 *	24.1
+ZA-A (10 μM)	1.650 ± 0.07 *	2.8	143.5 ± 4.94 *	12.7
+ZA-A (20 μM)	0.450 ± 0.08 *	10.3	48.5 ± 2.12 *	37.6
+ZA-B (10 μM)	1.900 ± 0.14 *	2.4	228.5 ± 2.23 *	8
+ZA-B (20 μM)	0.750 ± 0.07 *	6.2	141.5 ± 4.78 *	12.9
+ZA-C (10 μM)	4.000 ± 0.28	1.2	253.6 ± 5.16 *	7.2
+ZA-C (20 μM)	3.700 ± 0.56	1.3	221.8 ± 2.54 *	8.2
Vincristine	41.5 ± 0.74	1	14540 ± 719.13	1
+Verapamil (2.5 μM)	37.9 ± 0.64	1.1	370.81 ± 8.34 *	39.2
+ZA-A (10 μM)	6.450 ± 1.06 *	6.4	2187 ± 30.7 *	6.6
+ZA-A (20 μM)	3.450 ± 0.77 *	12	321.5 ± 3.53 *	45.2
+ZA-B (10 μM)	8.350 ± 1.17 *	5	2252 ± 11.31 *	6.5
+ZA-B (20 μM)	5.700 ± 0.98 *	7.3	1355.5 ± 30.41 *	10.7
+ZA-C (10 μM)	31.500 ± 2.47	1.3	2484 ± 55.15 *	5.9
+ZA-C (20 μM)	16.500 ± 0.71 *	2.5	971.5 ± 37.8 *	15

^1^ RF: Reversal fold; * *p* < 0.05 compared with substrate drugs transport with the tested compounds.

## Supplementary Materials

The following are available online. S01: References of the known compounds; Figures S1–S36: NMR spectra of compounds **1**–**4**.
