# Supplementary material for: Constituents and Anti-Multidrug Resistance Activity of Taiwanofungus camphoratus on Human Cervical Cancer Cells
Source: Molecules. 2019 Oct 16;24(20):3730. doi: 10.3390/molecules24203730 (PMC6833117; doi:10.3390/molecules24203730)

Supplementary Materials

Constituents and Anti-Multidrug Resistance Activity of *Taiwanofungus camphoratus* on Human Cervical Cancer Cells

Hsin-Yi Hung [1], Chin-Chuan Hung [2], Jun-Weil Liang [3], Chin-Fu Chen [4], Hung-Yi Chen [2], Po-Chuen Shieh [5], Ping-Chung Kuo [1,*] and Tian-Shung Wu [1,5,*]

[1] School of Pharmacy, College of Medicine, National Cheng Kung University, Tainan 701, Taiwan; z10308005@email.ncku.edu.tw
[2] Department of Pharmacy, College of Pharmacy, China Medical University, Taichung 402, Taiwan; cc0206hung@gmail.com (C.-C.H.), hungyi@mail.cmu.edu.tw (H.-Y.C.)
[3] Department of Chemistry, National Cheng Kung University, Tainan 701, Taiwan; p77625@hotmail.com
[4] Department of Life Sciences, National Cheng Kung University, Tainan 701, Taiwan; chinfu9999@gmail.com
[5] Department of Pharmacy, College of Pharmacy and Health Care, Tajen University, Pingtung 907, Taiwan; pochuen@tajen.edu.tw (P.-C.S.)
* Correspondence: tswu@mail.ncku.edu.tw (T.-S.W.); z10502016@email.ncku.edu.tw (P.-C.K.); Tel.: +886-2353535-65333 (T.-S.W.); Tel.: +886-6-2353535-6806 (P.-C.K.)

Contents

S01. References of the known compounds.

Fig. S01. ESI-MS spectrum of Camphoratin K (**1**)

Fig. S02. HRMS spectrum of Camphoratin K (**1**)

Fig. S03. IR spectrum of Camphoratin K (**1**).

Fig. S04. ^{1}H NMR spectrum of Camphoratin K (**1**)

Fig. S05. ^{13}C and DEPT NMR spectrum of Camphoratin K (**1**)

Fig. S06. COSY spectrum of Camphoratin K (**1**)

Fig. S07. HSQC spectrum of Camphoratin K (**1**)

Fig. S08. HMBC spectrum of Camphoratin K (**1**)

Fig. S09. NOESY spectrum of Camphoratin K (**1**)

Fig. S10. ESI-MS spectrum of Camphoratin N (**2**)

Fig. S11. HRMS spectrum of Camphoratin N (**2**)

Fig. S12. IR spectrum of Camphoratin N (**2**)

Fig. S13. ^{1}H NMR spectrum of Camphoratin N (**2**)

Fig. S14. ^{13}C and DEPT NMR spectrum of Camphoratin N (**2**)

Fig. S15. COSY spectrum of Camphoratin N (**2**)

Fig. S16. HSQC spectrum of Camphoratin N (**2**)

Fig. S17. HMBC spectrum of Camphoratin N (**2**)

Fig. S18. NOESY spectrum of Camphoratin N (**2**)

Fig. S19. ESI-MS spectrum of Benzocamphorin G (**3**).

Fig. S20. HRMS spectrum of Benzocamphorin G (**3**)

Fig. S21. IR spectrum of Benzocamphorin G (**3**).

Fig. S22. ^{1}H NMR spectrum of Benzocamphorin G (**3**)

Fig. S23. ^{13}C and DEPT NMR spectrum of Benzocamphorin G (**3**)

Fig. S24. COSY spectrum of Benzocamphorin G (**3**)

Fig. S25. HSQC spectrum of Benzocamphorin G (**3**)

Fig. S26. HMBC spectrum of Benzocamphorin G (**3**)

Fig. S27. NOESY spectrum of Benzocamphorin G (**3**)

Fig. S28. ESI-MS spectrum of Benzocamphorin I (**4**).

Fig. S29. HRMS spectrum of Benzocamphorin I (**4**)

Fig. S30. IR spectrum of Benzocamphorin I (**4**).

Fig. S31. ^{1}H NMR spectrum of Benzocamphorin I (**4**)

Fig. S32. ^{13}C and DEPT NMR spectrum of Benzocamphorin I (**4**)

Fig. S33. COSY spectrum of Benzocamphorin I (**4**)

Fig. S34. HSQC spectrum of Benzocamphorin I (**4**)

Fig. S35. HMBC spectrum of Benzocamphorin I (**4**)

Fig. S36. NOESY spectrum of Benzocamphorin I (**4**)

S01. References of the known compounds.

Methyl antcinate A (**5**)[1],

Antcins A (**6**), C (**12**), and K (**18**), zhankuic acid A methyl ester (**7**), zhankuic acids A (**8**), B (**11**), C (**9**) , camphoratins E (**13**) and F (**14**), methyl antcinate H (**15**), antcamphin D (**17**), antcamphins A (**19**), B (**16**), ergosterol (**24**), ergosterol peroxide (**25**), camphoratin I (**26**), sesamin (**27**) and 4-hydroxysesamin (**28**), antrocamphins A (**30**) and B (**29**), methyl 3,4,5-trimethoxybenzoate (**33**),benzocamphorins C (**37**), D (**44**), E (**43**), methyl 2,5-dimethoxy-3,4-methylenedioxybenzoate (**38**), 4,7-dimethoxyl-5-methyl-1,3-benzodioxole (**40**), 2,2,5,5-tetramethoxyl-3,4,3,4-bimethylenedioxyl-6,6-dimethylbiphenyl (**42**)[2,3]

Zhankuic acid D (**10**)[4]

1-Hydroxy-*p*-menth-3-en-2-one (**20**)[5]

Coenzyme Q (**22**)[6]

4-Acetylantroquinonol B (**23**)[7]

Benzocamphorin F (**31**)[8]

Benzocamphorin H (**32**)[9]

Methyl 2,3,4,5-tetramethoxy benzoate (**34**), 1-methyl-2,3,4,5-trimethoxy benzene (**35**)[10]

2,3,6-Trimethoxy-5-methylphenol (**36**), 2,3-(methylenedioxy)-4-methyl-5-methylphenol (**41**)[11]

4,5-Dimethoxy-6-methyl-1,3-benzodioxole (**39**)[12]

Tetracanyl ferulate (**45**)[13]

Reference:

1. Tsai, W.C.; Rao, Y.K.; Lin, S.S.; Chou, M.Y.; Shen, Y.T.; Wu, C.H.; Geethangili, M.; Yang, C.C.; Tzeng, Y.M. Methylantcinate A induces tumor specific growth inhibition in oral cancer cells via Bax-mediated mitochondrial apoptotic pathway. *Bioorg Med Chem Lett* **2010**, *20*, 6145-6148, doi:10.1016/j.bmcl.2010.08.006.
2. Shi, L.S.; Chao, C.H.; Shen, D.Y.; Chan, H.H.; Chen, C.H.; Liao, Y.R.; Wu, S.J.; Leu, Y.L.; Shen, Y.C.; Kuo, Y.H., et al. Biologically active constituents from the fruiting body of Taiwanofungus camphoratus. *Bioorg Med Chem* **2011**, *19*, 677-683, doi:10.1016/j.bmc.2010.10.032.
3. Wu, S.J.; Leu, Y.L.; Chen, C.H.; Chao, C.H.; Shen, D.Y.; Chan, H.H.; Lee, E.J.; Wu, T.S.; Wang, Y.H.; Shen, Y.C., et al. Camphoratins A-J, potent cytotoxic and anti-inflammatory triterpenoids from the fruiting body of

Taiwanofungus camphoratus. *J Nat Prod* **2010**, *73*, 1756-1762, doi:10.1021/np1002143.
4. Yang, S.-W.; Shen, Y.-C.; Chen, C.-H. Steroids and triterpenoids of Antodia cinnamomea—A fungus parasitic on Cinnamomum micranthum. *Phytochemistry* **1996**, *41*, 1389-1392, doi:https://doi.org/10.1016/0031- 9422(95)00767-9.
5. Matsuura, T.; Suga, T. Oxidation of Terpene Compounds with t-Butyl Chromate. VIII.1 The Oxidation of α-Terpinene2. *The Journal of Organic Chemistry* **1965**, *30*, 518-520, doi:10.1021/jo01013a052.
6. Wu, M.D.; Cheng, M.J.; Wang, W.Y.; Huang, H.C.; Yuan, G.F.; Chen, J.J.; Chen, I.S.; Wang, B.C. Antioxidant activities of extracts and metabolites isolated from the fungus Antrodia cinnamomea. *Nat Prod Res* **2011**, *25*, 1488- 1496, doi:10.1080/14786410903132563.
7. Chen, M.-C.; Cho, T.-Y.; Kuo, Y.-H.; Lee, T.-H. Meroterpenoids from a Medicinal Fungus Antrodia cinnamomea. *Journal of Natural Products* **2017**, *80*, 2439-2446, doi:10.1021/acs.jnatprod.7b00223.
8. Lee, C.L.; Huang, C.H.; Wang, H.C.; Chuang, D.W.; Wu, M.J.; Wang, S.Y.; Hwang, T.L.; Wu, C.C.; Chen, Y.L.; Chang, F.R., et al. First total synthesis of antrocamphin A and its analogs as anti-inflammatory and anti-platelet aggregation agents. *Org Biomol Chem* **2011**, *9*, 70-73, doi:10.1039/c0ob00616e.
9. Liao, Y.-R.; Kuo, P.-C.; Huang, S.-C.; Liang, J.-W.; Wu, T.-S. An efficient total synthesis of Benzocamphorin H and its anti-inflammatory activity. *Tetrahedron Letters* **2012**, *53*, 6202-6204, doi:https://doi.org/10.1016/j.tetlet.2012.08.138.
10. Syper, L.; Kloc, K.; Mz.xl; lochowski, J. Synthesis of ubiquinone and menaquinone analogues by oxidative demethylation of alkenylhydroquinone ethers with argentic oxide or ceric ammonium nitrat. *Tetrahedron* **1980**, *36*, 123-129, doi:https://doi.org/10.1016/0040-4020(80)85034-4.
11. Chiu, H.-L.; Wu, J.-H.; Tung, Y.-T.; Lee, T.-H.; Chien, S.-C.; Kuo, Y.-H. Triterpenoids and Aromatics from Derris laxiflora. *Journal of Natural Products* **2008**, *71*, 1829-1832, doi:10.1021/np800253s.
12. Liu, S.Y.W., W.C.; Tsou, W.L.; Kuo, M. T. Compounds from antrodia camphorata for inhibiting the growth of cancer tumor cells. US2008/103195,2008,A1, 2008.
13. Shi, Y.G.; Zhu, Y.J.; Shao, S.Y.; Zhang, R.R.; Wu, Y.; Zhu, C.M.; Liang, X.R.; Cai, W.Q. Alkyl Ferulate Esters as Multifunctional Food Additives: Antibacterial Activity and Mode of Action against Escherichia coli in Vitro.*J. Agric Food Chem* **2018**, *66*, 12088-12101, doi:10.1021/acs.jafc.8b04429.

Fig. S01. ESI-MS spectrum of Camphoratin K (**1**)

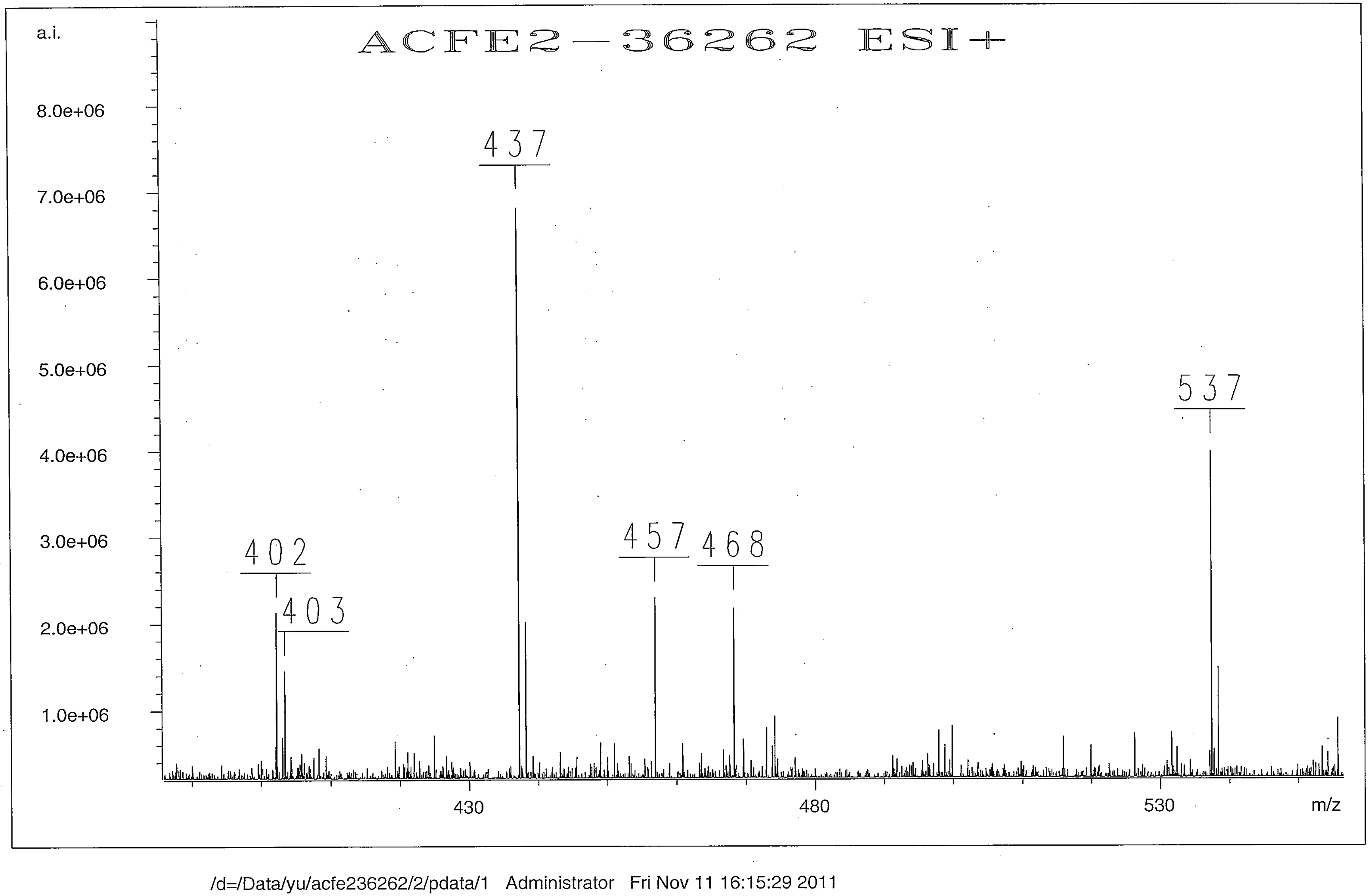

Fig. S02. HRMS spectrum of Camphoratin K (**1**)

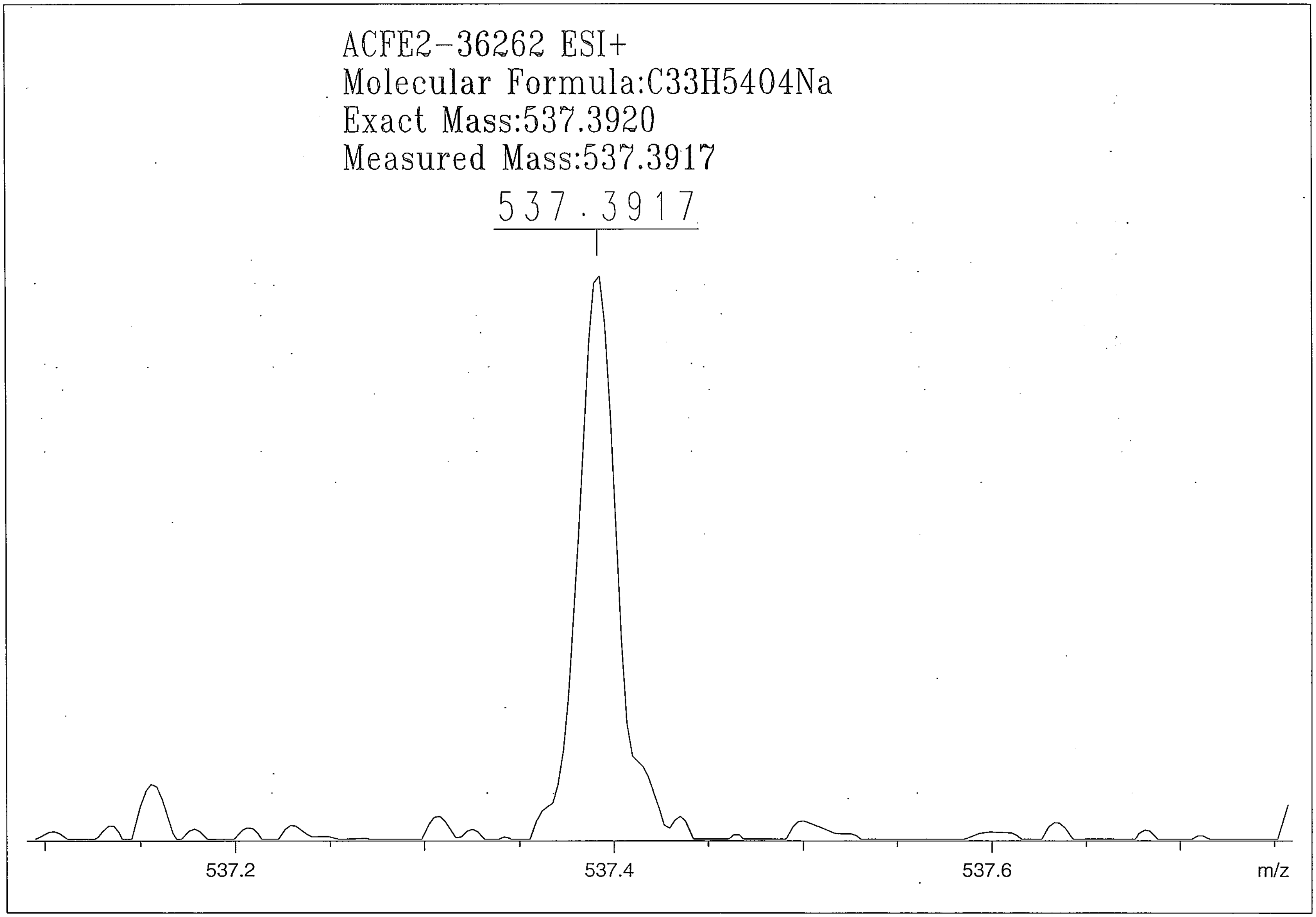

/d=/Data/yu/acfe236262/1/pdata/1 Administrator Fri Nov 11 16:17:45 2011

Fig. S03. IR spectrum of Camphoratin K (**1**).

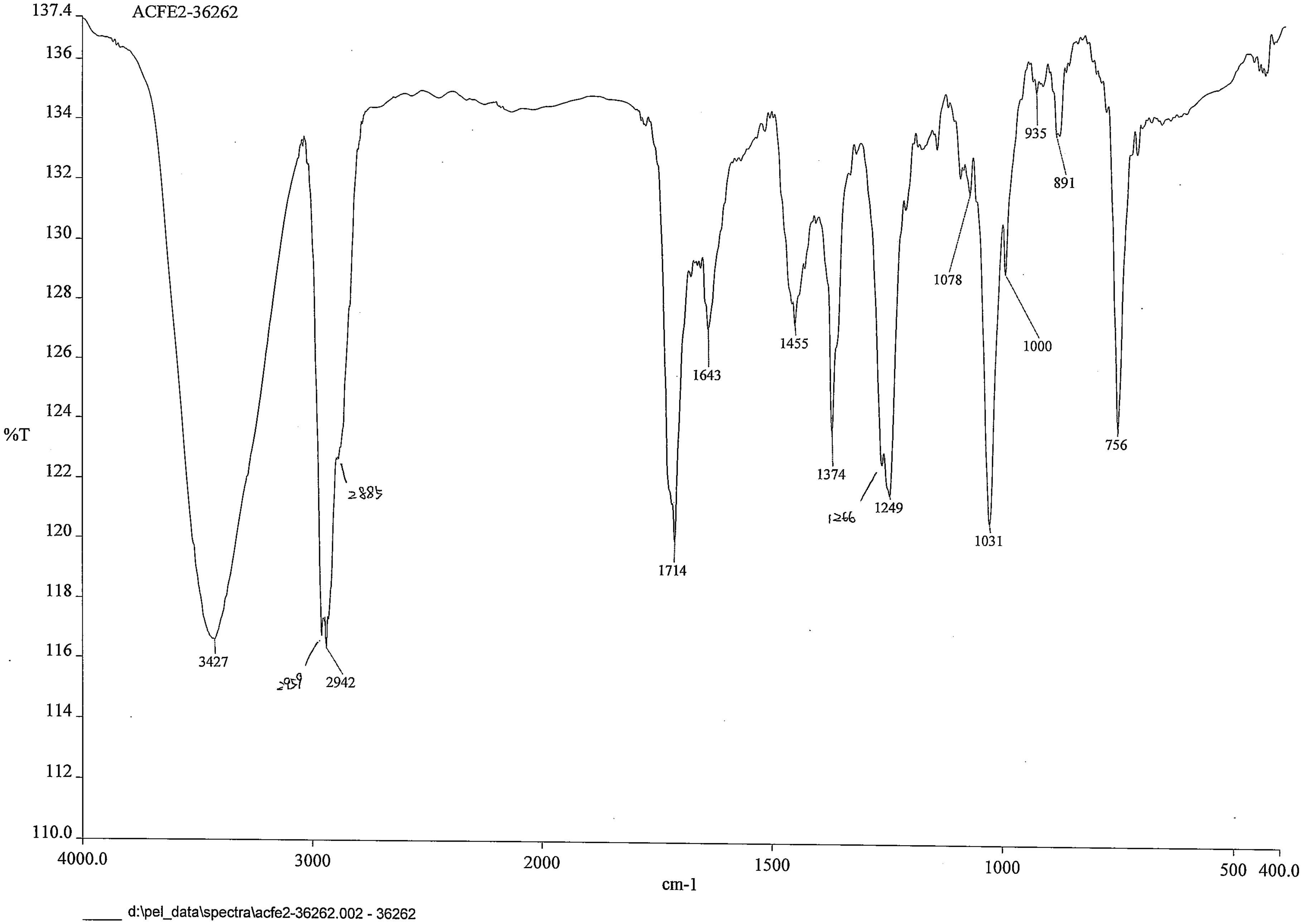

Fig. S04. ^{1}H NMR spectrum of Camphoratin K (**1**)

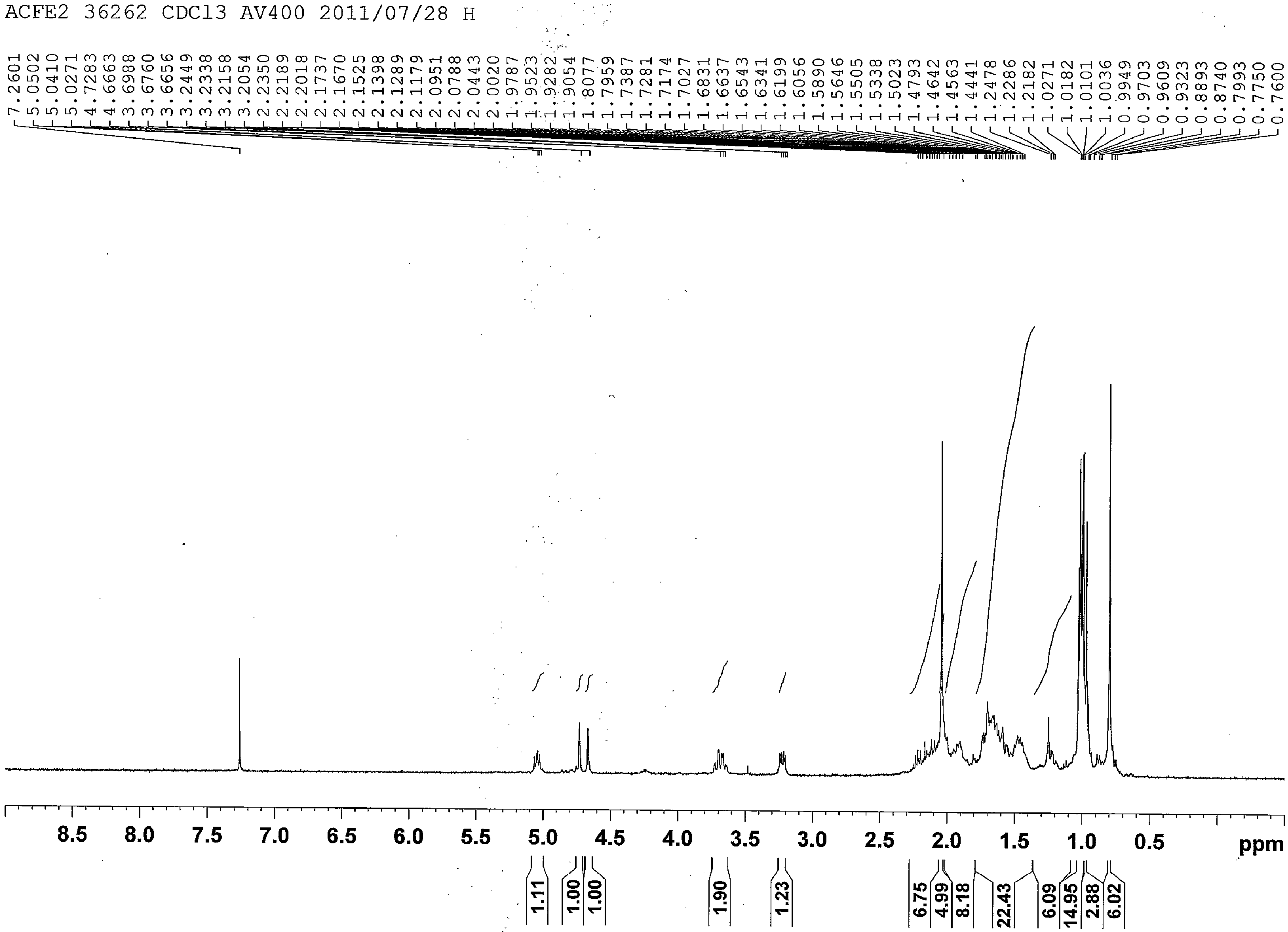

Fig. S05. ^{13}C and DEPT NMR spectrum of Camphoratin K (**1**)

ACFE2 36262 CDCl3 AV400 2011/07/28 13C

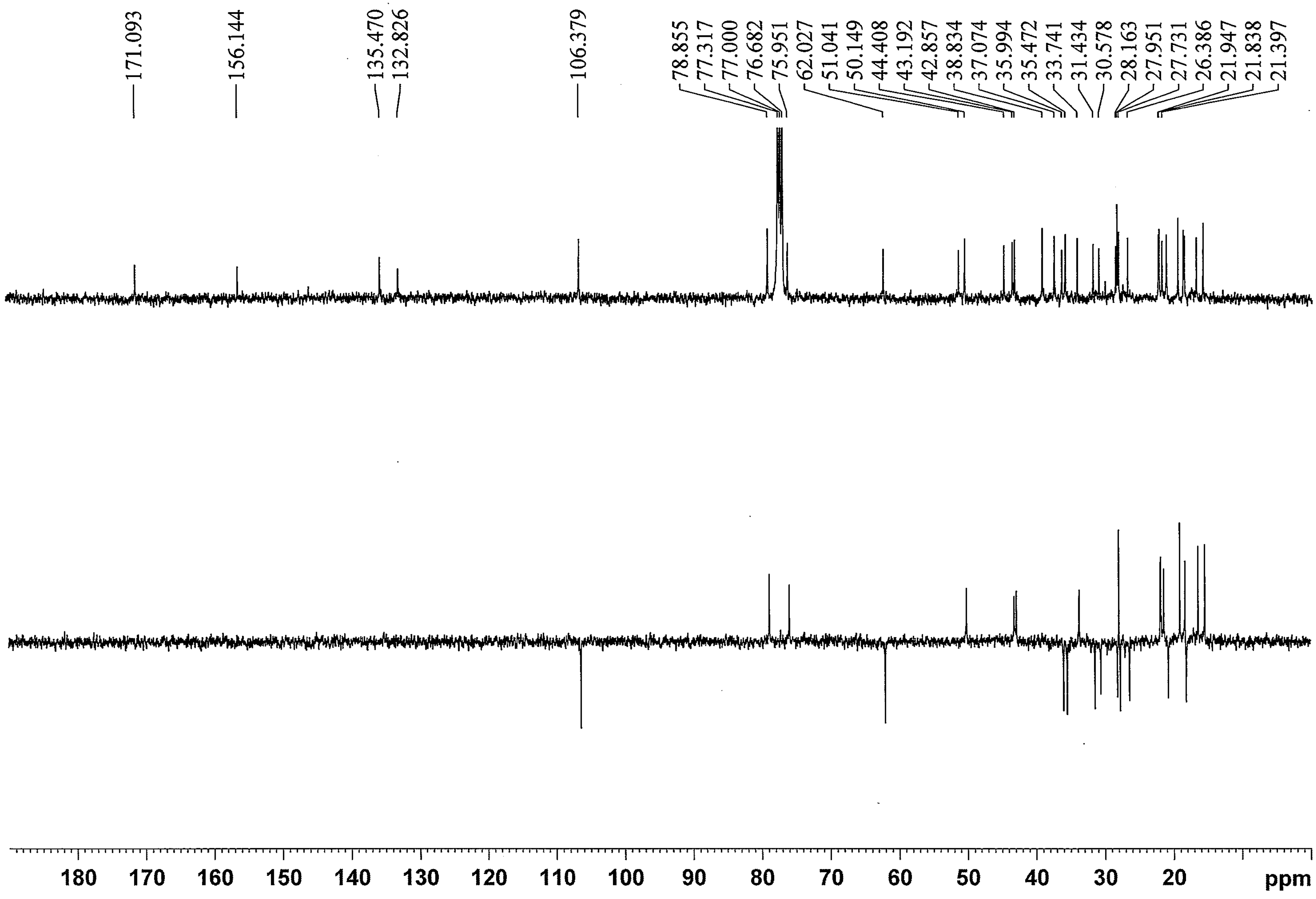

Fig. S06. COSY spectrum of Camphoratin K (**1**)

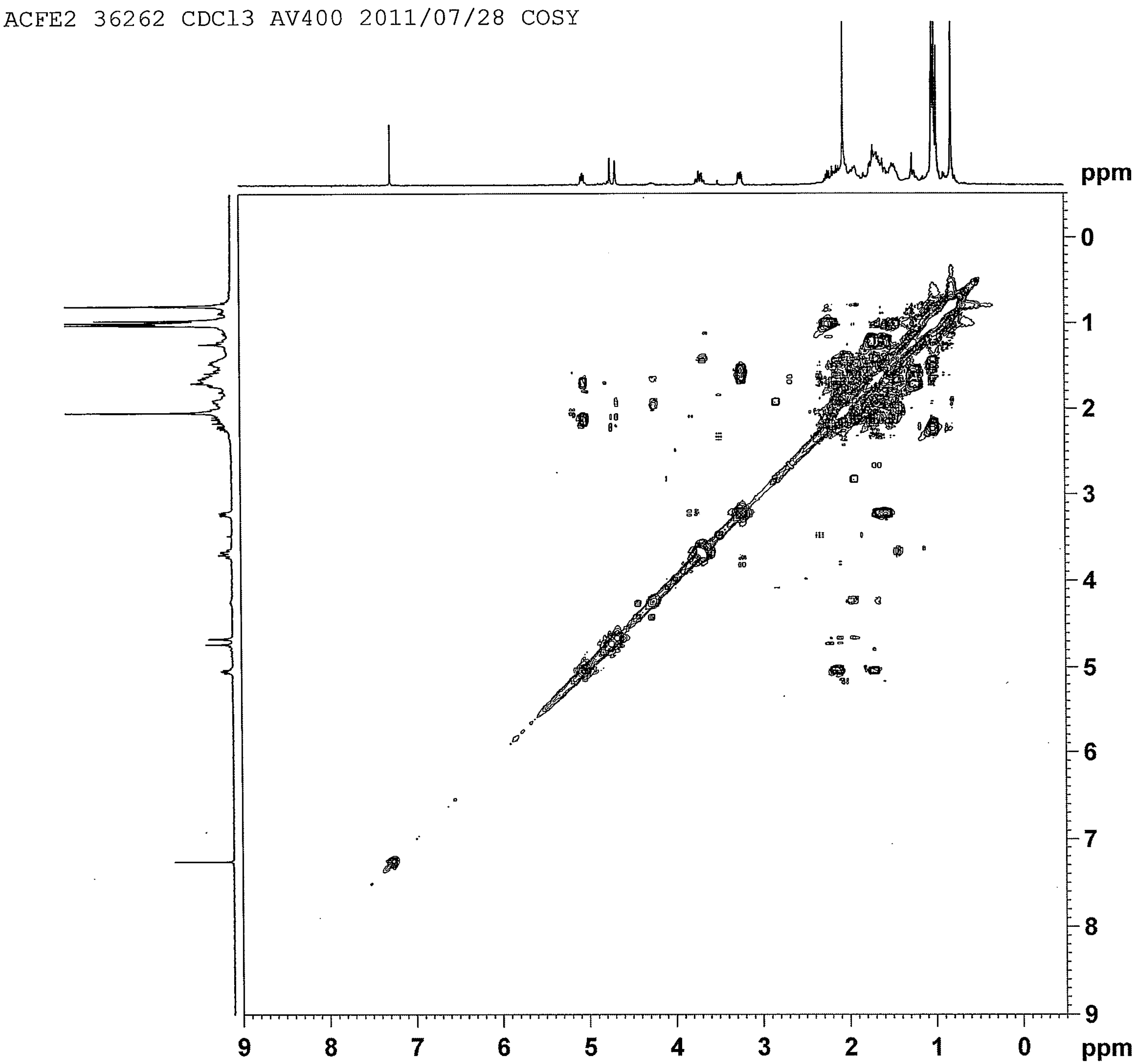

Fig. S07. HSQC spectrum of Camphoratin K (**1**)

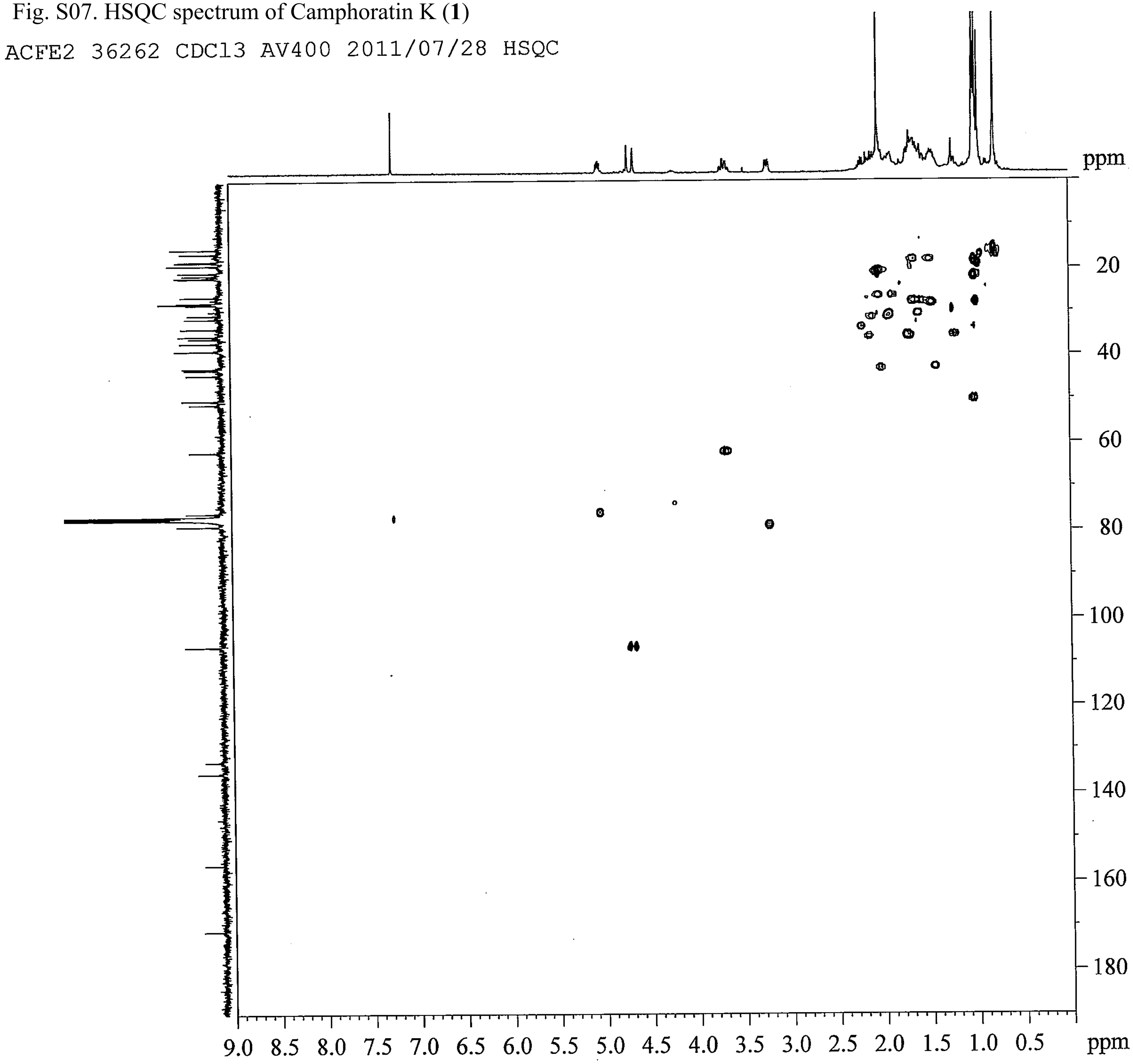

Fig. S08. HMBC spectrum of Camphoratin K (**1**)

ACFE2 36262 CDCl3 AV400 2011/07/28 HMBC

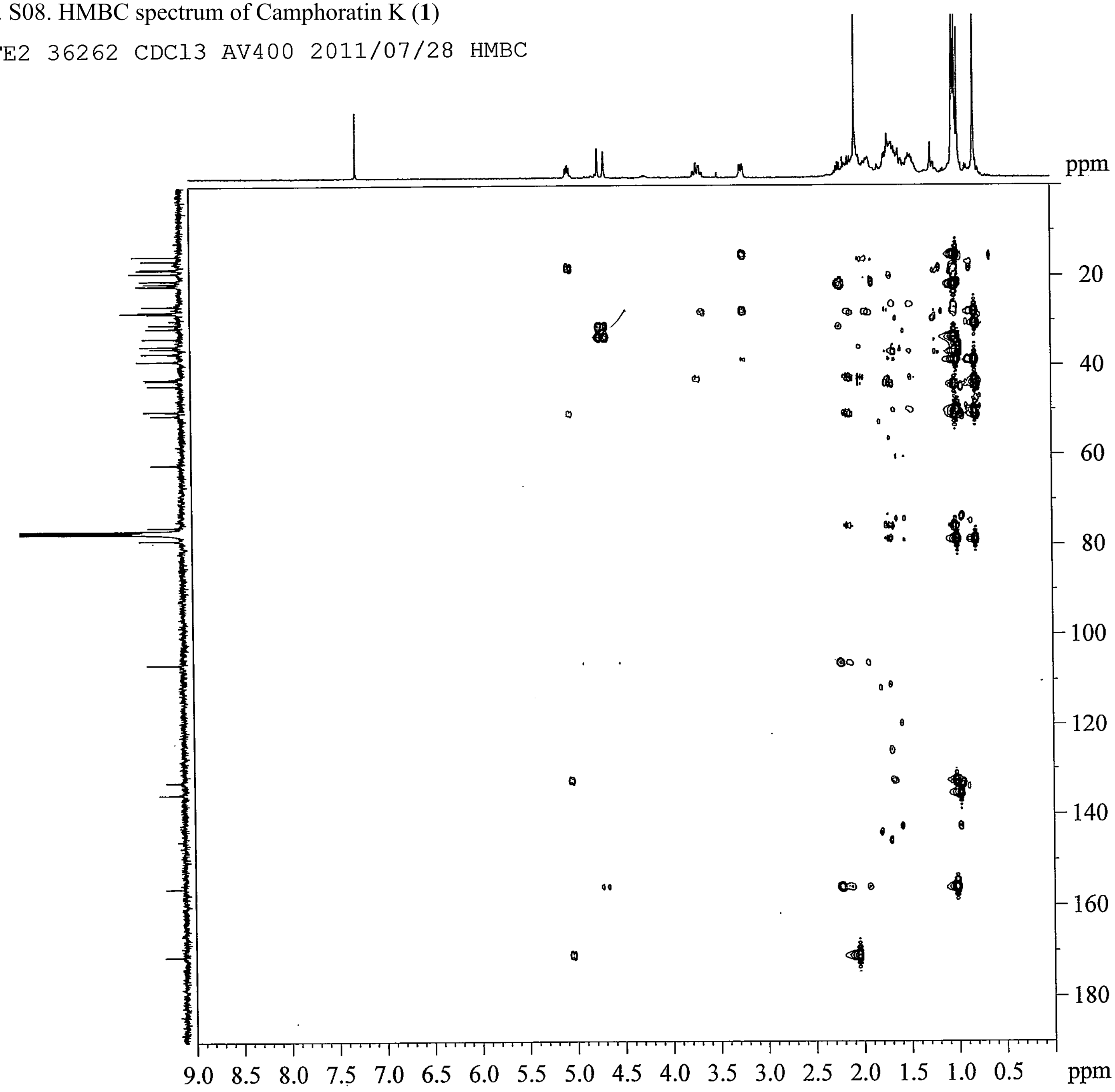

Fig. S09. NOESY spectrum of Camphoratin K (**1**)

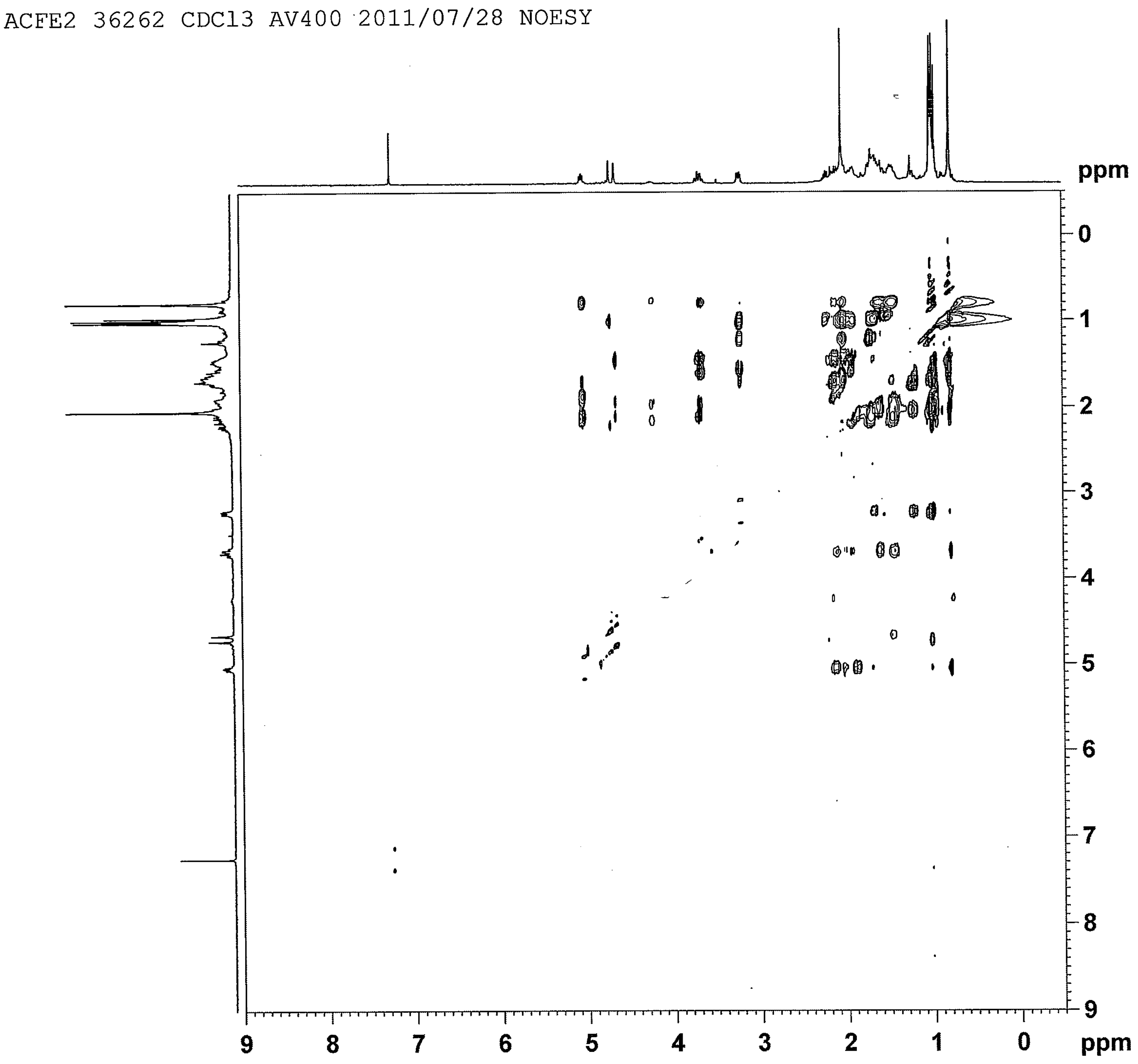

Fig. S10. ESI-MS spectrum of Camphoratin N (**2**)

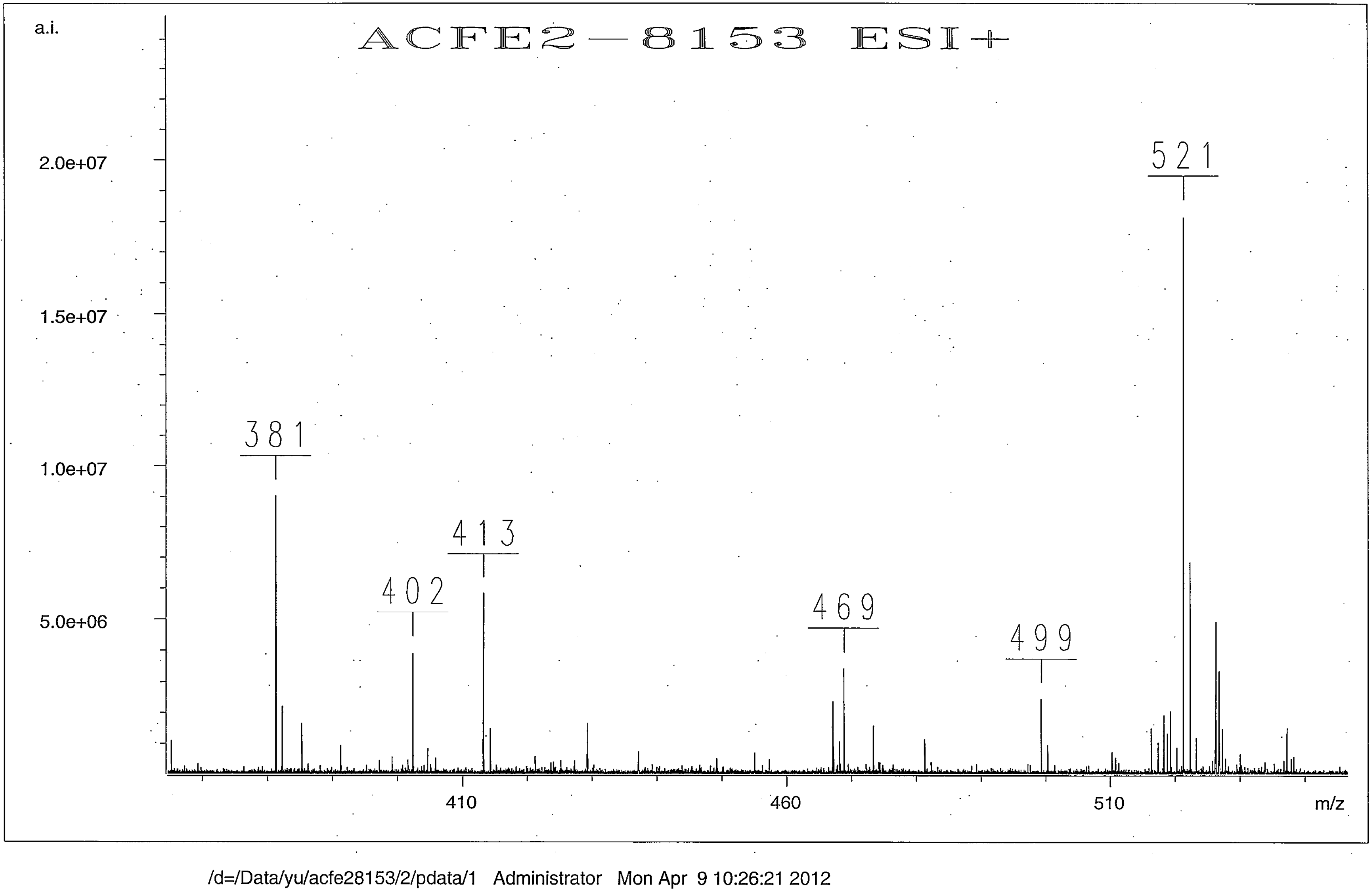

Fig. S11. HRMS spectrum of Camphoratin N (**2**)

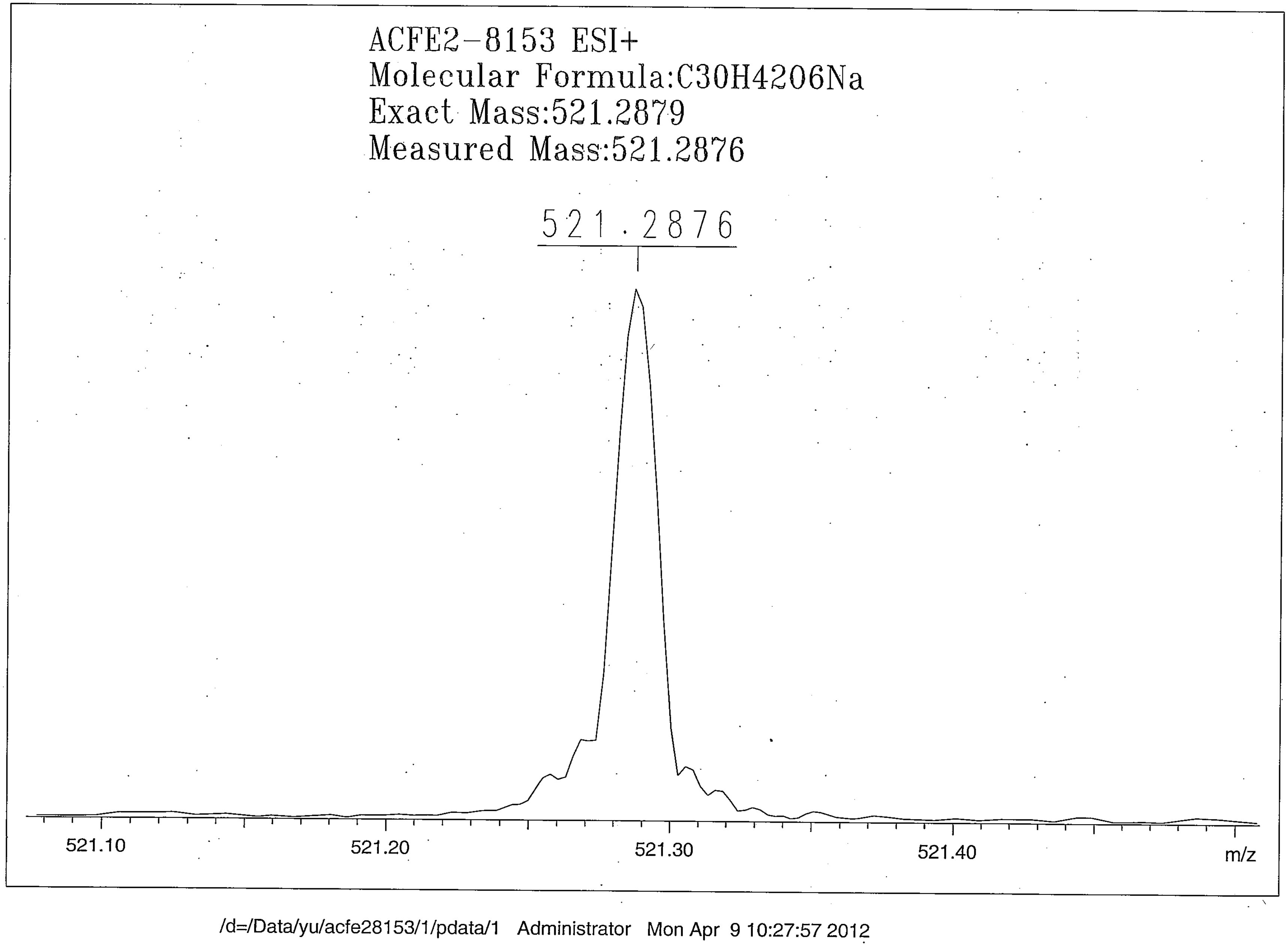

Fig. S12. IR spectrum of Camphoratin N (**2**)

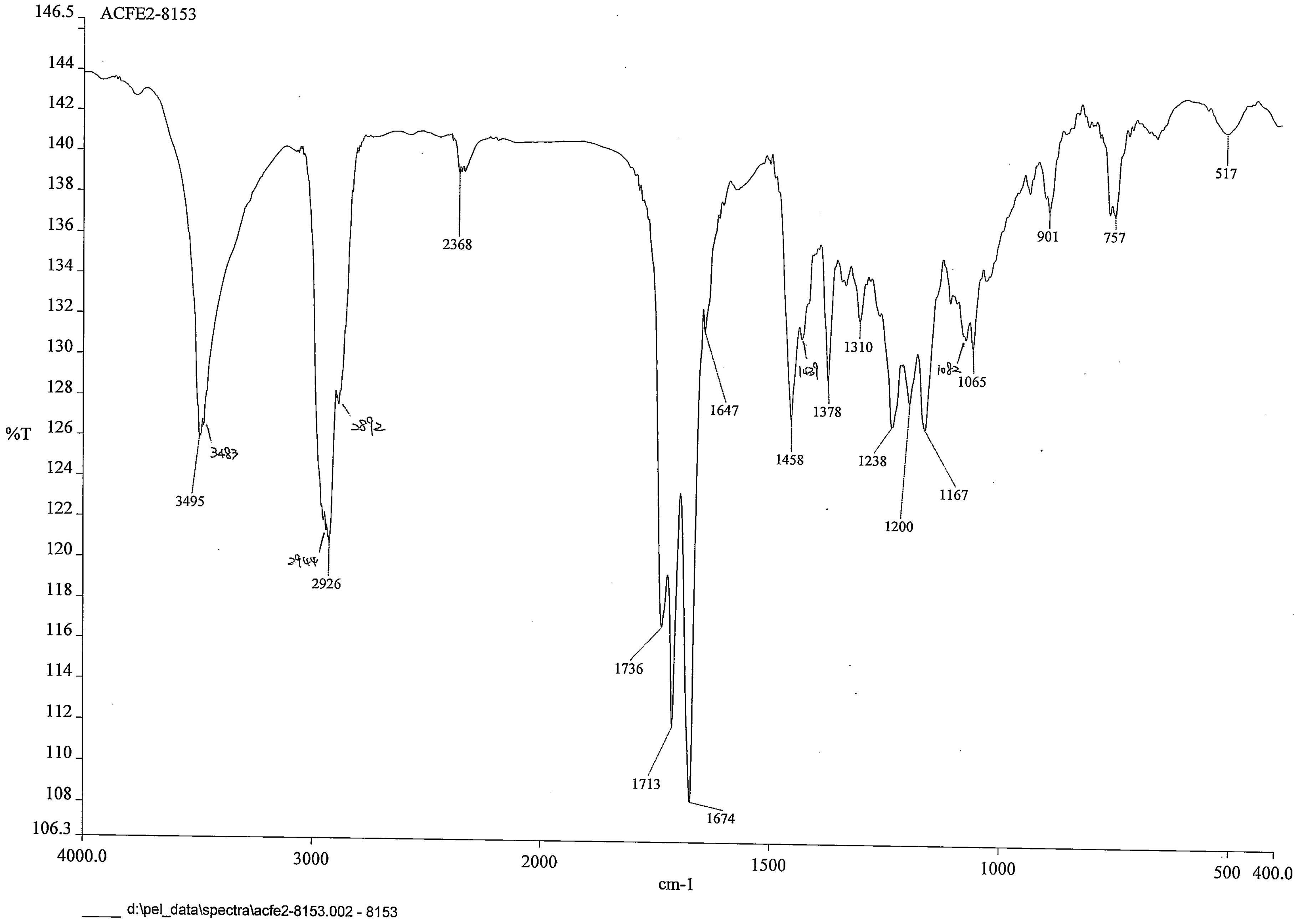

Fig. S13. ^{1}H NMR spectrum of Camphoratin N (**2**)

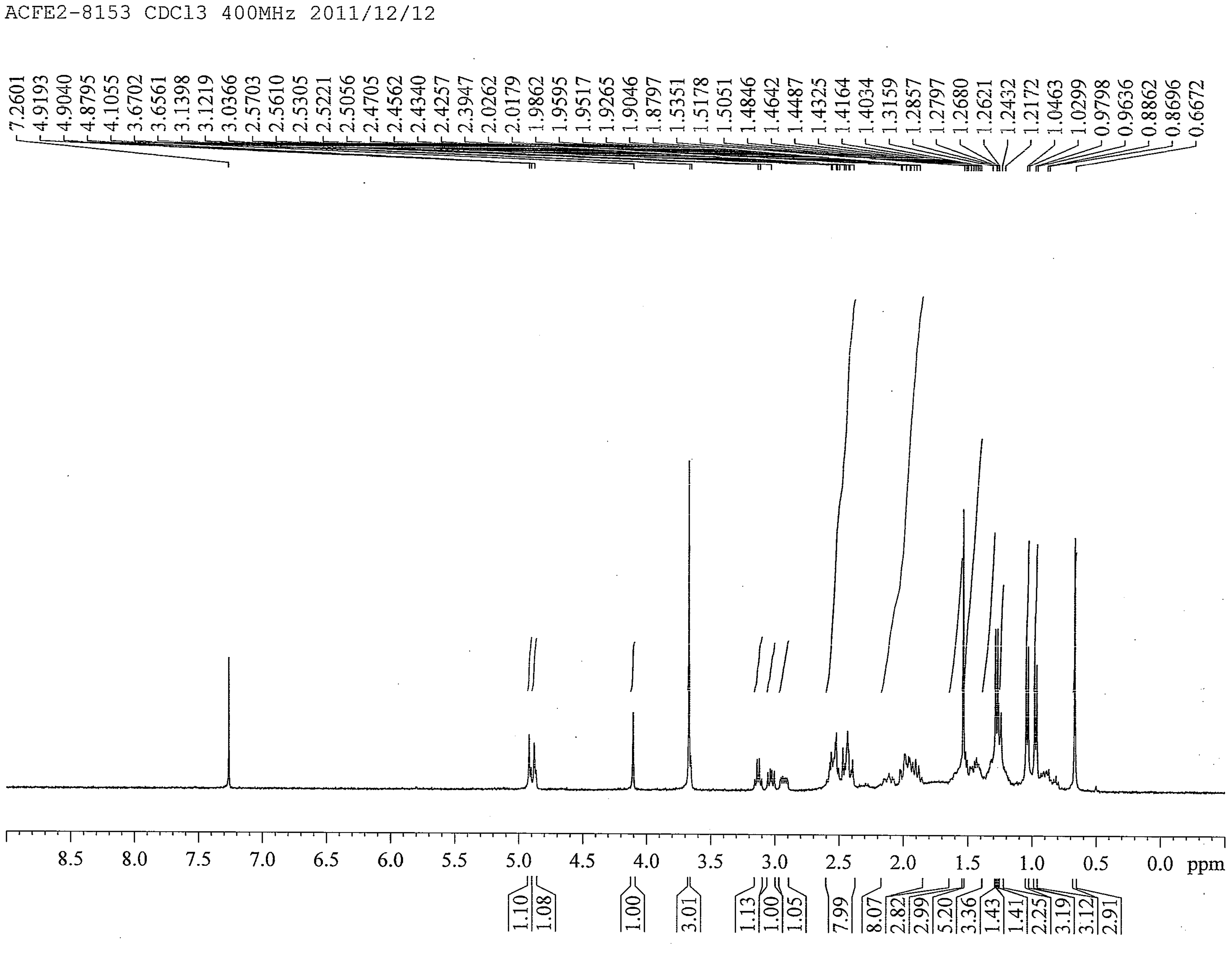

Fig. S14. ^{13}C and DEPT NMR spectrum of Camphoratin N (**2**)

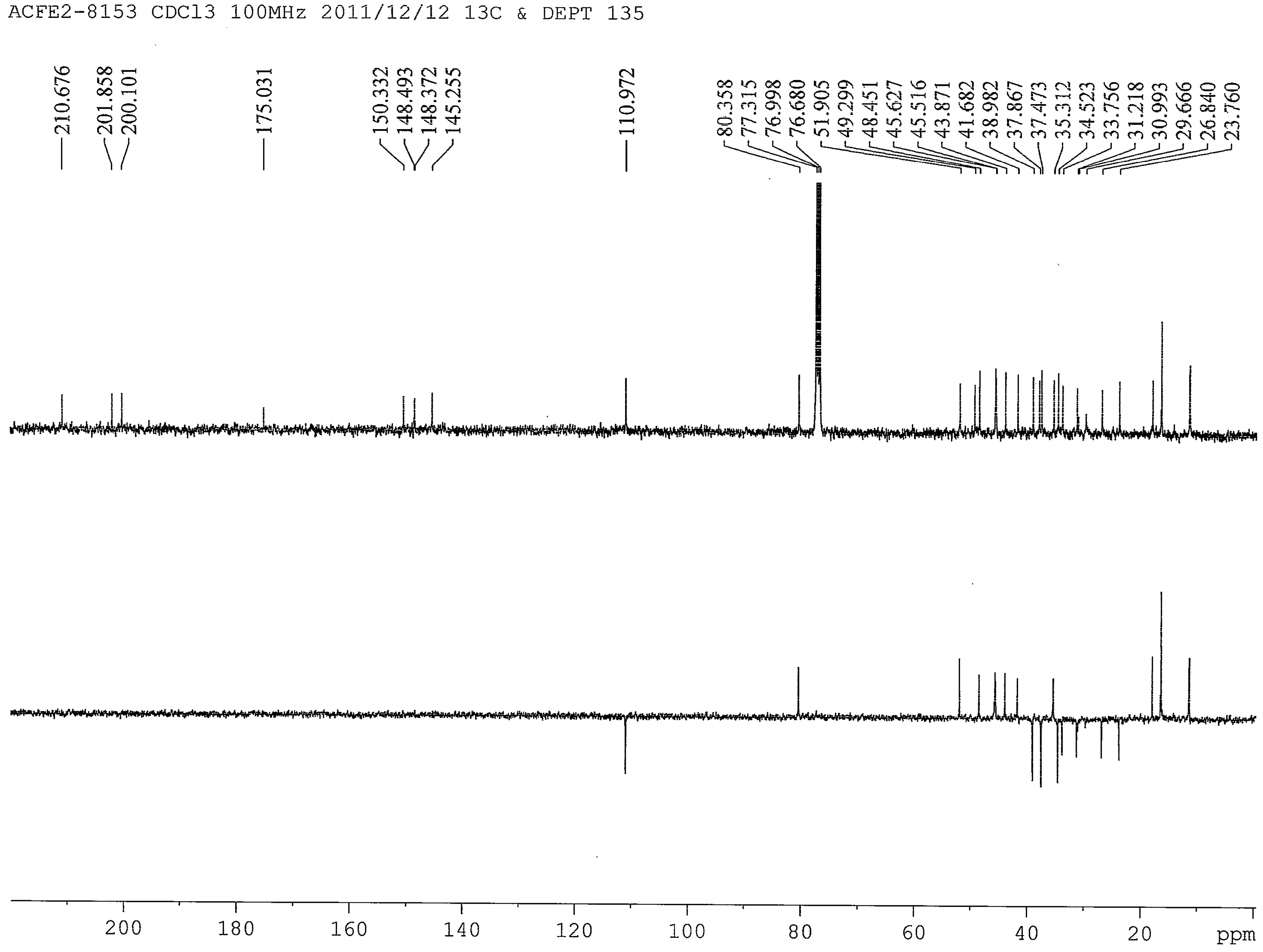

Fig. S15. COSY spectrum of Camphoratin N (**2**)

ACFE2-8153 CDCl3 400MHz 2011/12/12 COSY

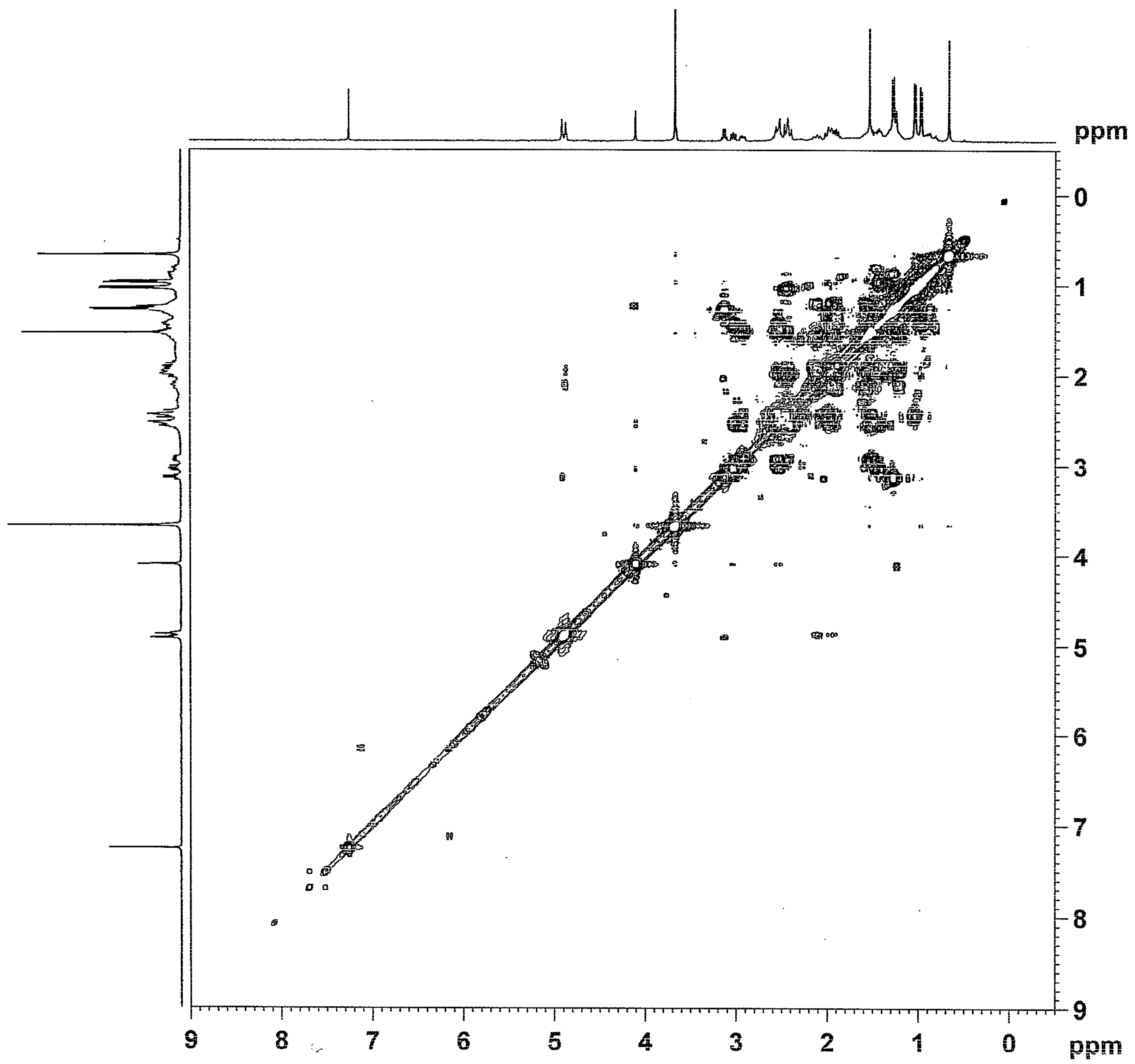

Fig. S16. HSQC spectrum of Camphoratin N (**2**)

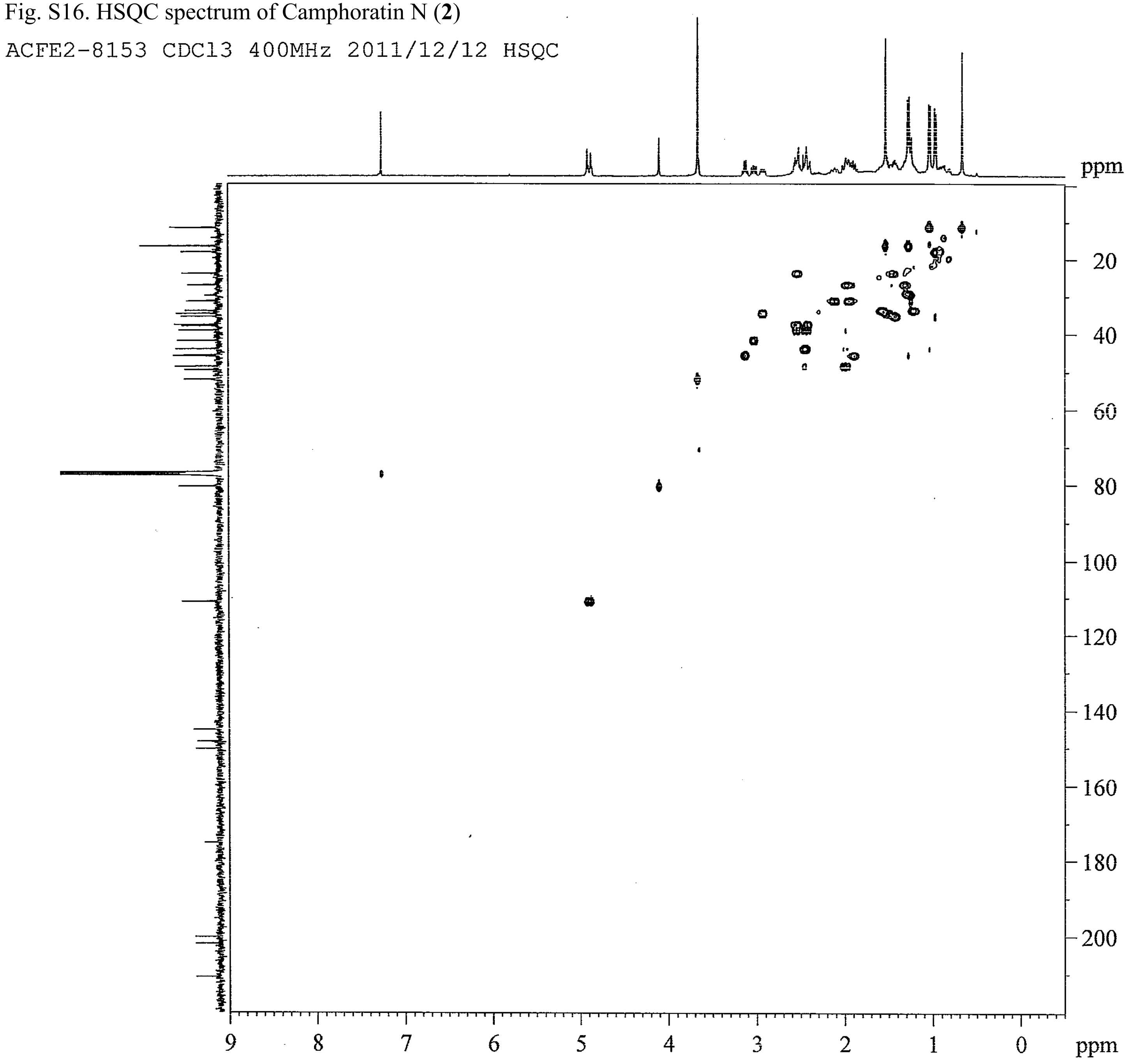

Fig. S17. HMBC spectrum of Camphoratin N (**2**)

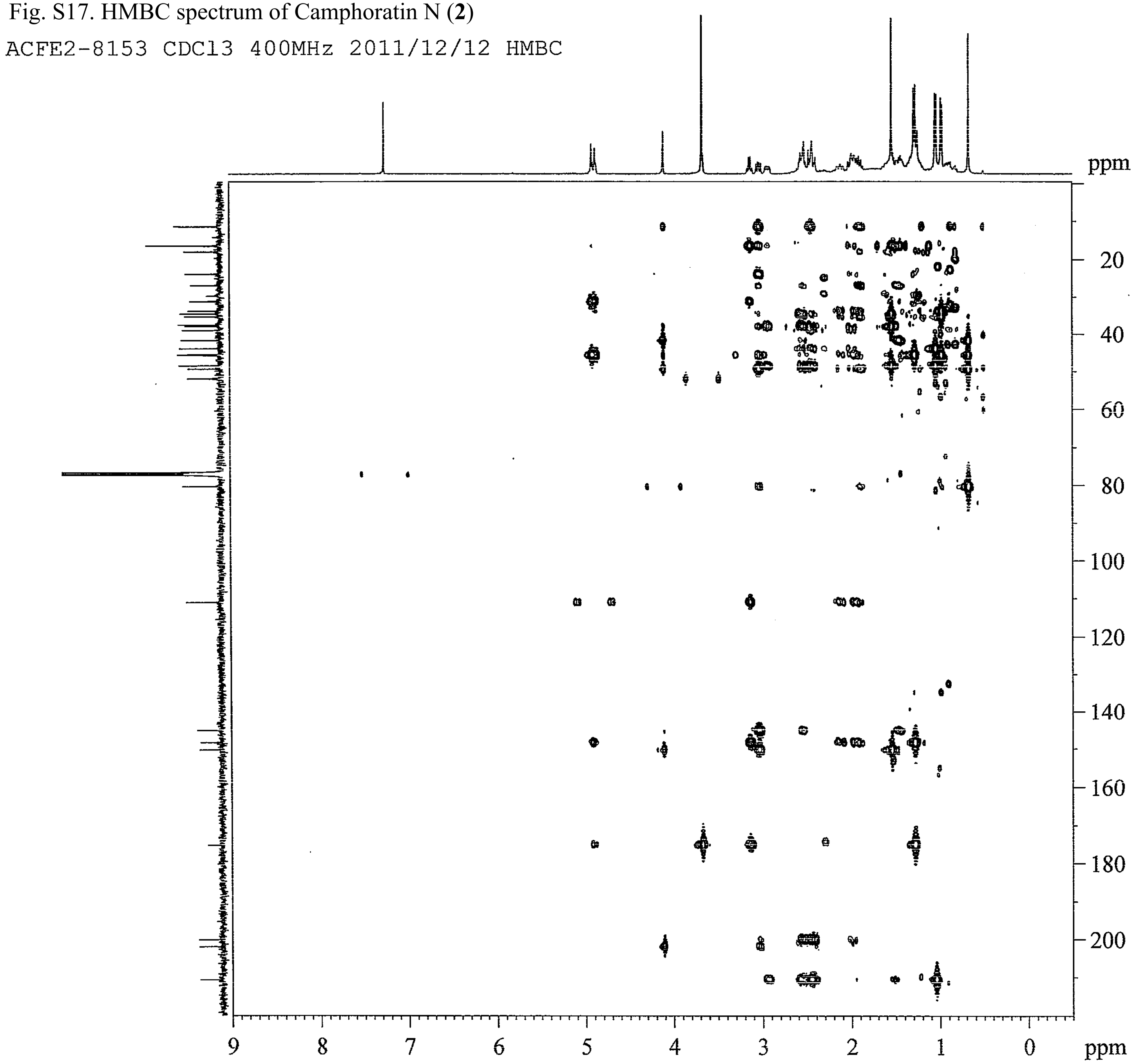

Fig. S18. NOESY spectrum of Camphoratin N (**2**)

ACFE2-8153 CDCl3 400MHz 2011/12/12 NOESY

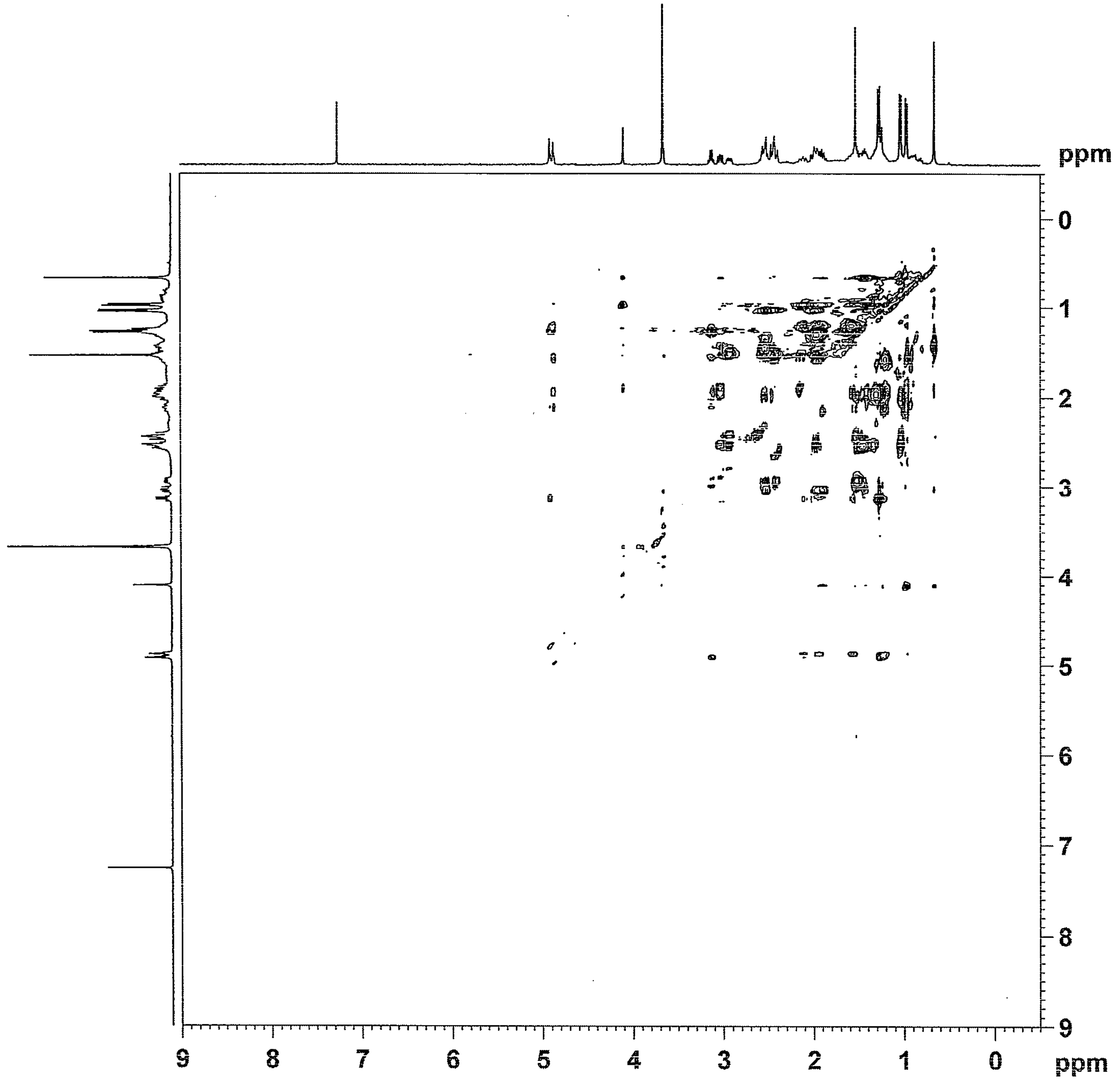

Fig. S19. ESI-MS spectrum of Benzocamphorin G (**3**)

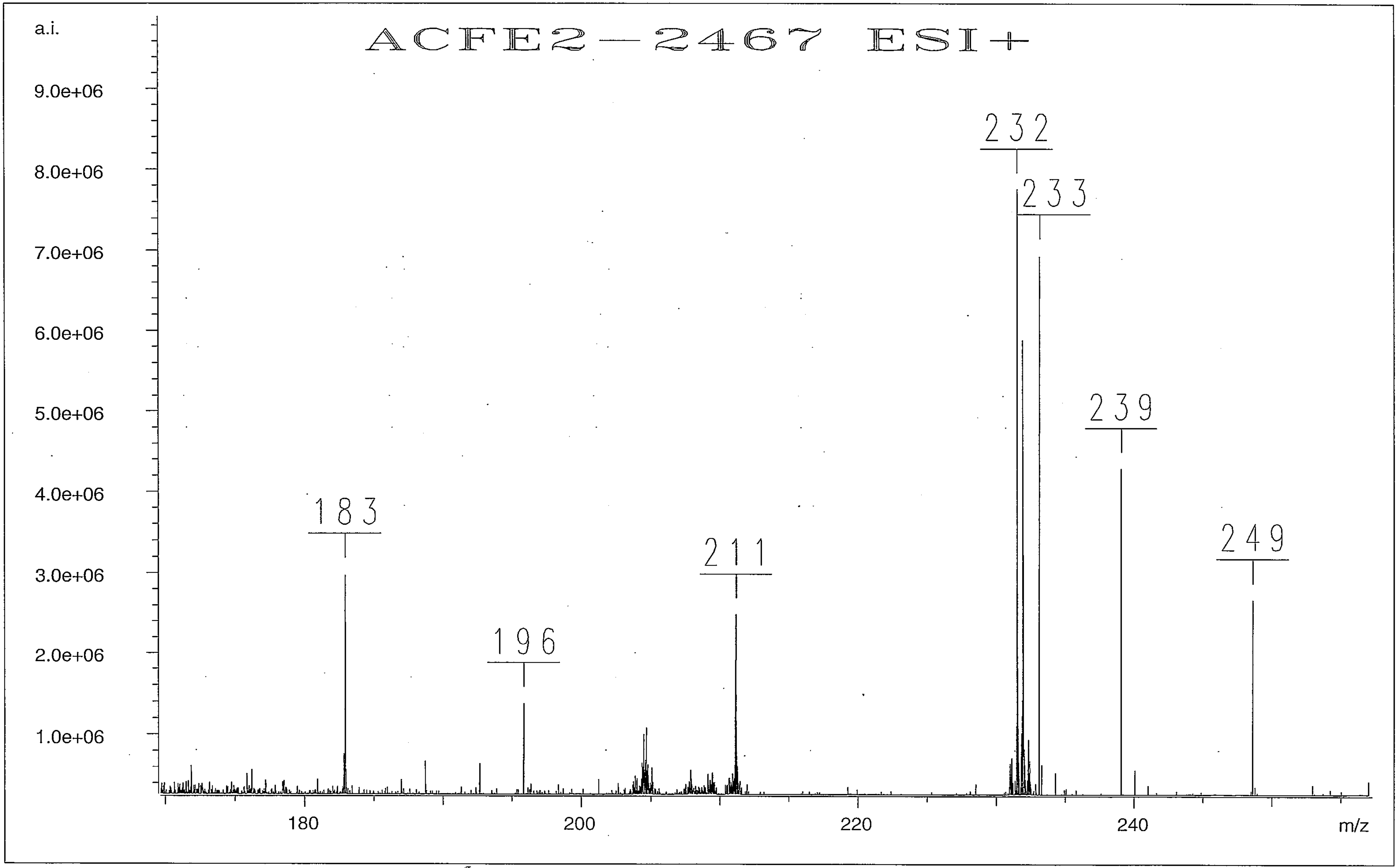

/d=/Data/yu/acfe22467/1/pdata/1 Administrator Fri Nov 11 15:42:39 2011

Fig. S20. HRMS spectrum of Benzocamphorin G (**3**)

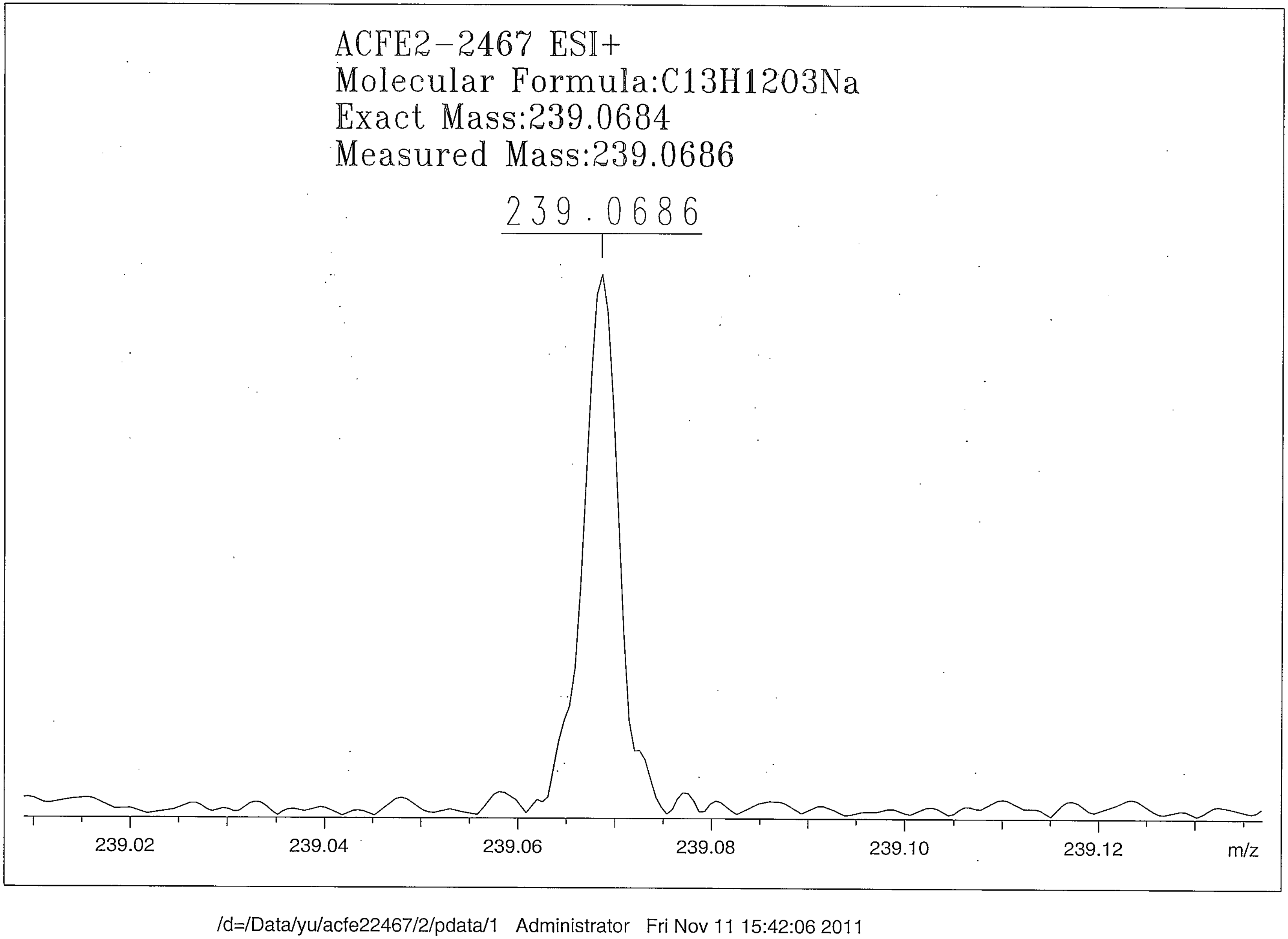

Fig. S21. IR spectrum of Benzocamphorin G (**3**).

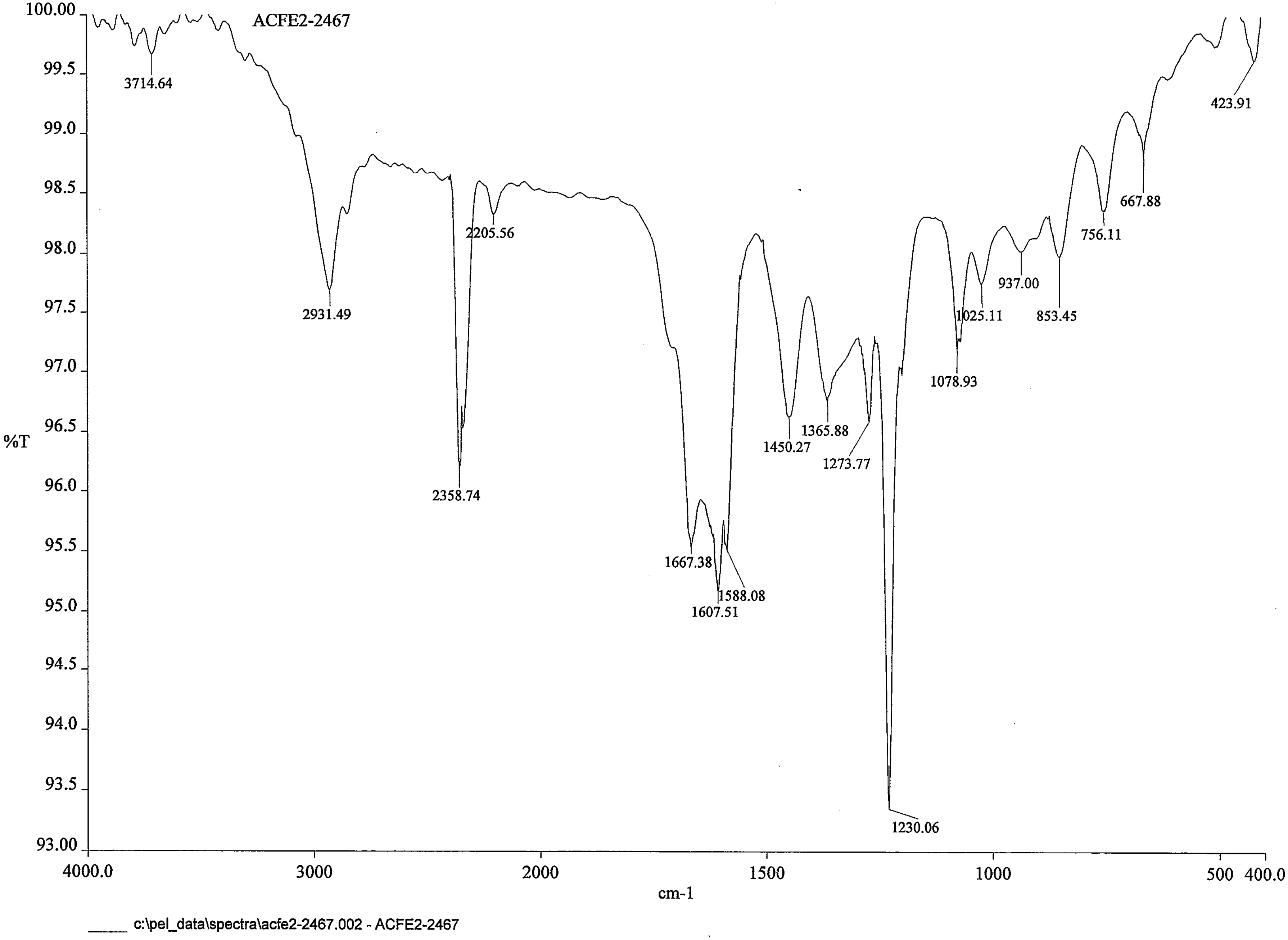

Fig. S22. ^{1}H NMR spectrum of Benzocamphorin G (**3**)

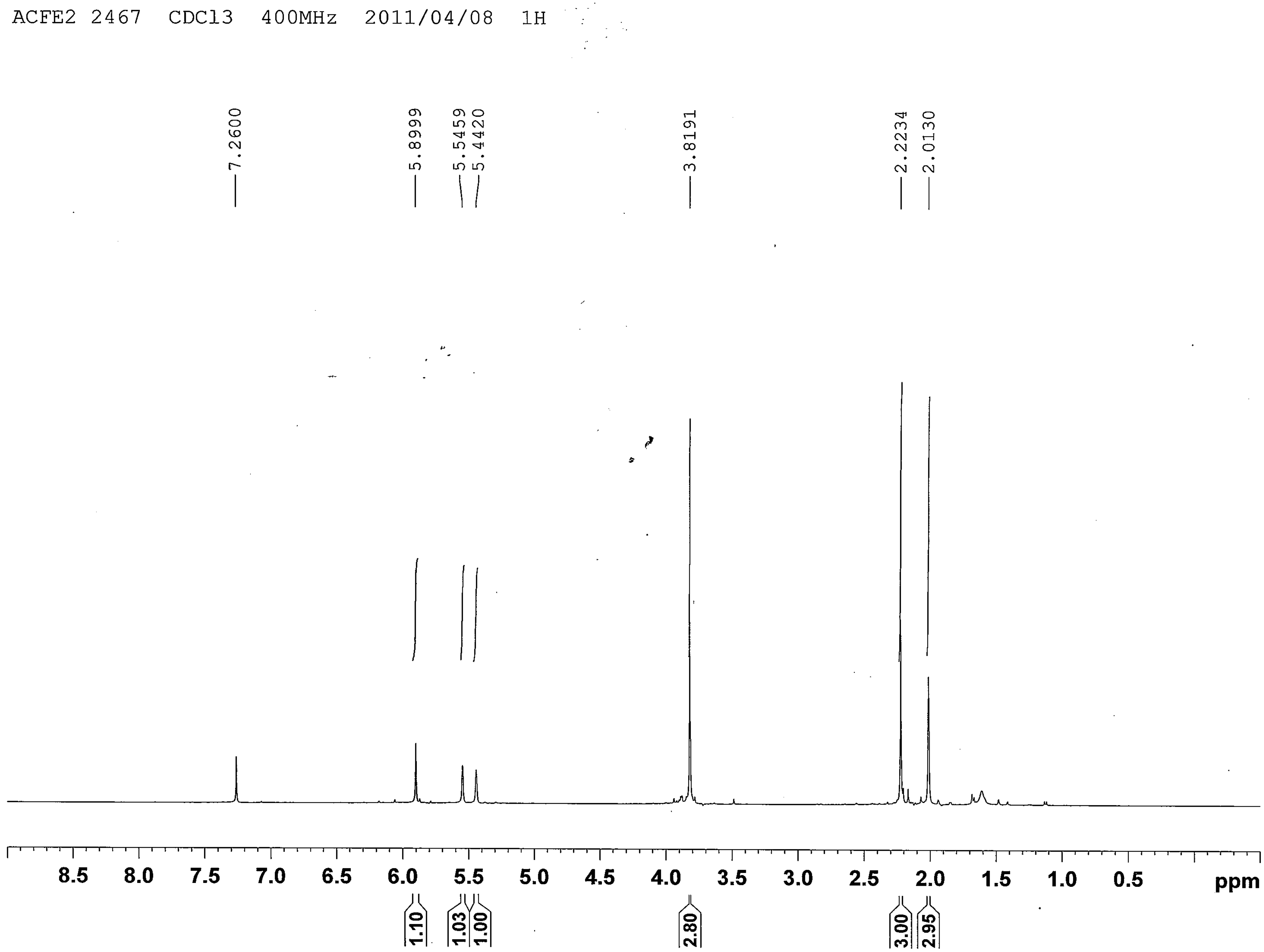

Fig. S23. ^{13}C and DEPT NMR spectrum of Benzocamphorin G (**3**)

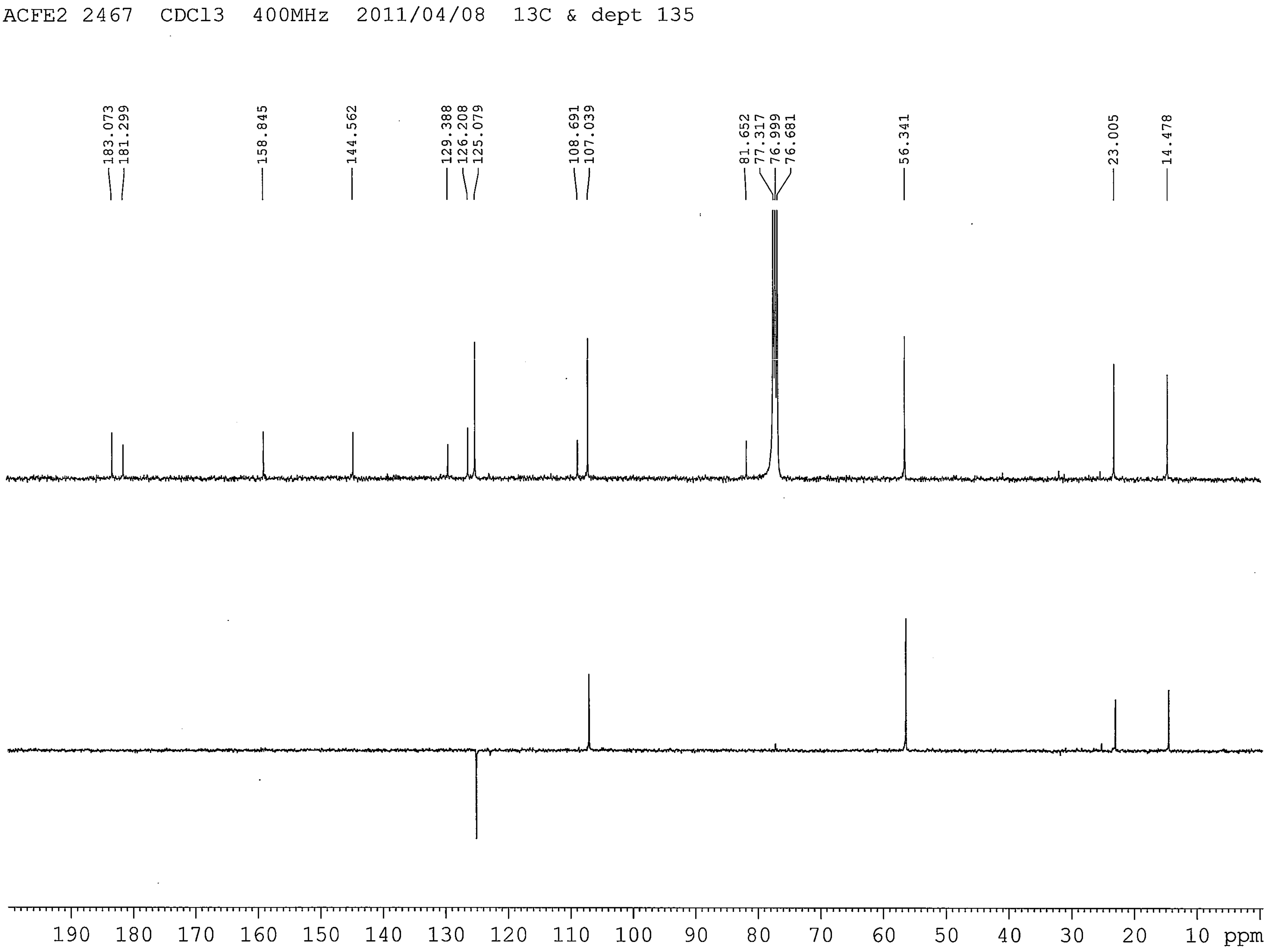

Fig. S24. COSY spectrum of Benzocamphorin G (**3**)

ACFE2 2467 CDCl3 400MHz 2011/04/08 COSY

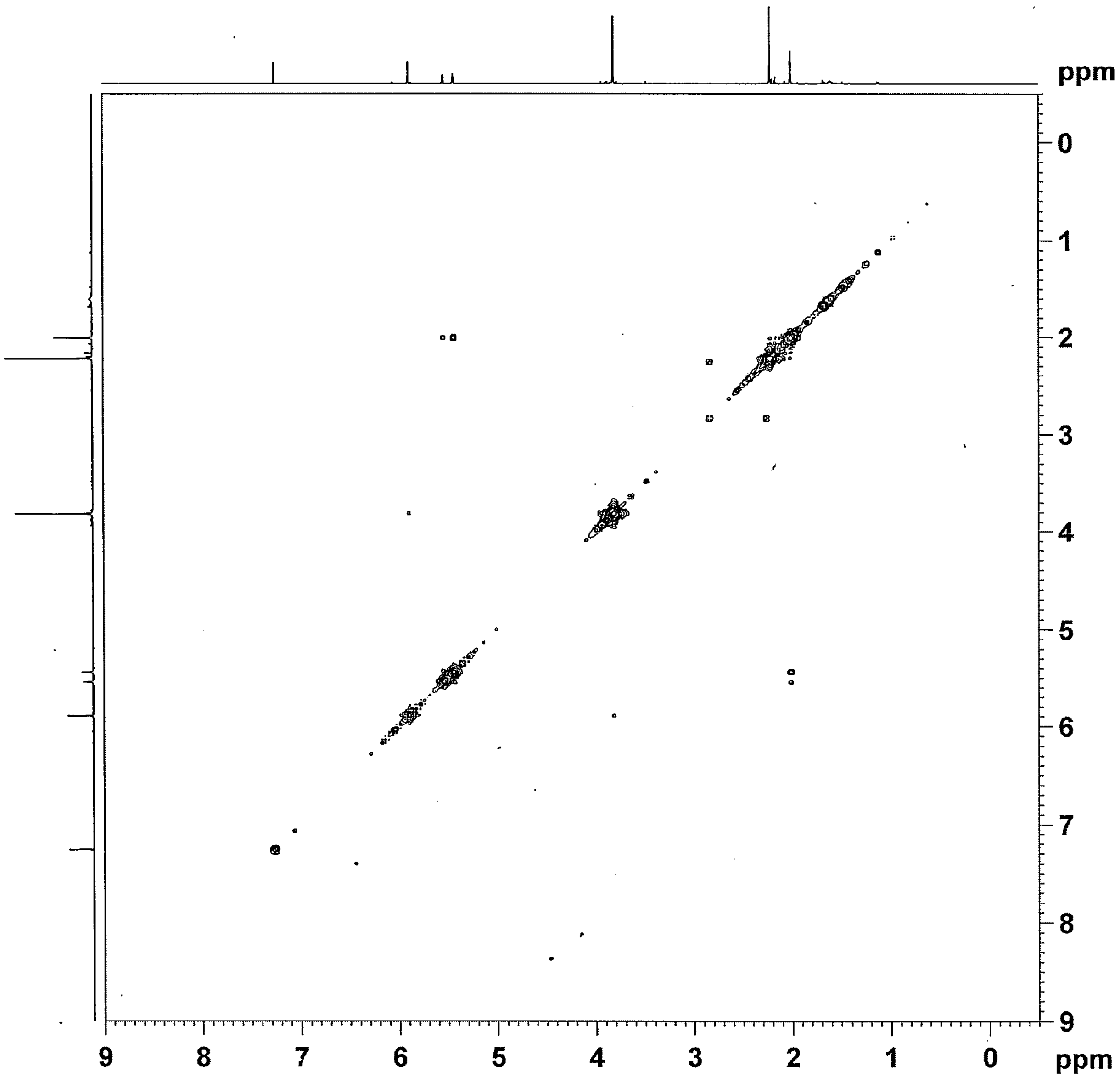

Fig. S25. HSQC spectrum of Benzocamphorin G (**3**)

ACFE2 2467 CDCl3 400MHz 2011/04/08 HSQC

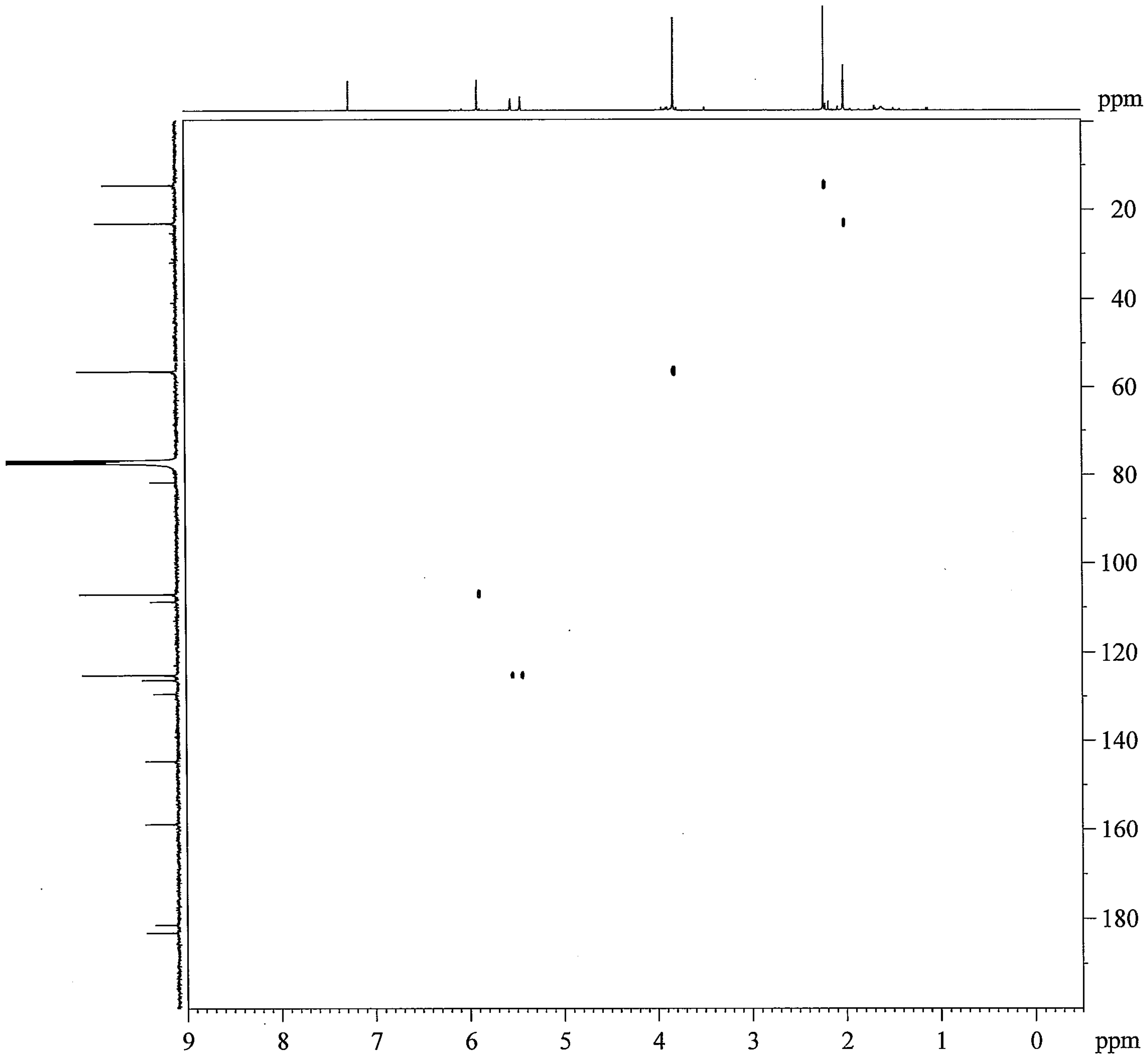

Fig. S26. HMBC spectrum of Benzocamphorin G (**3**)

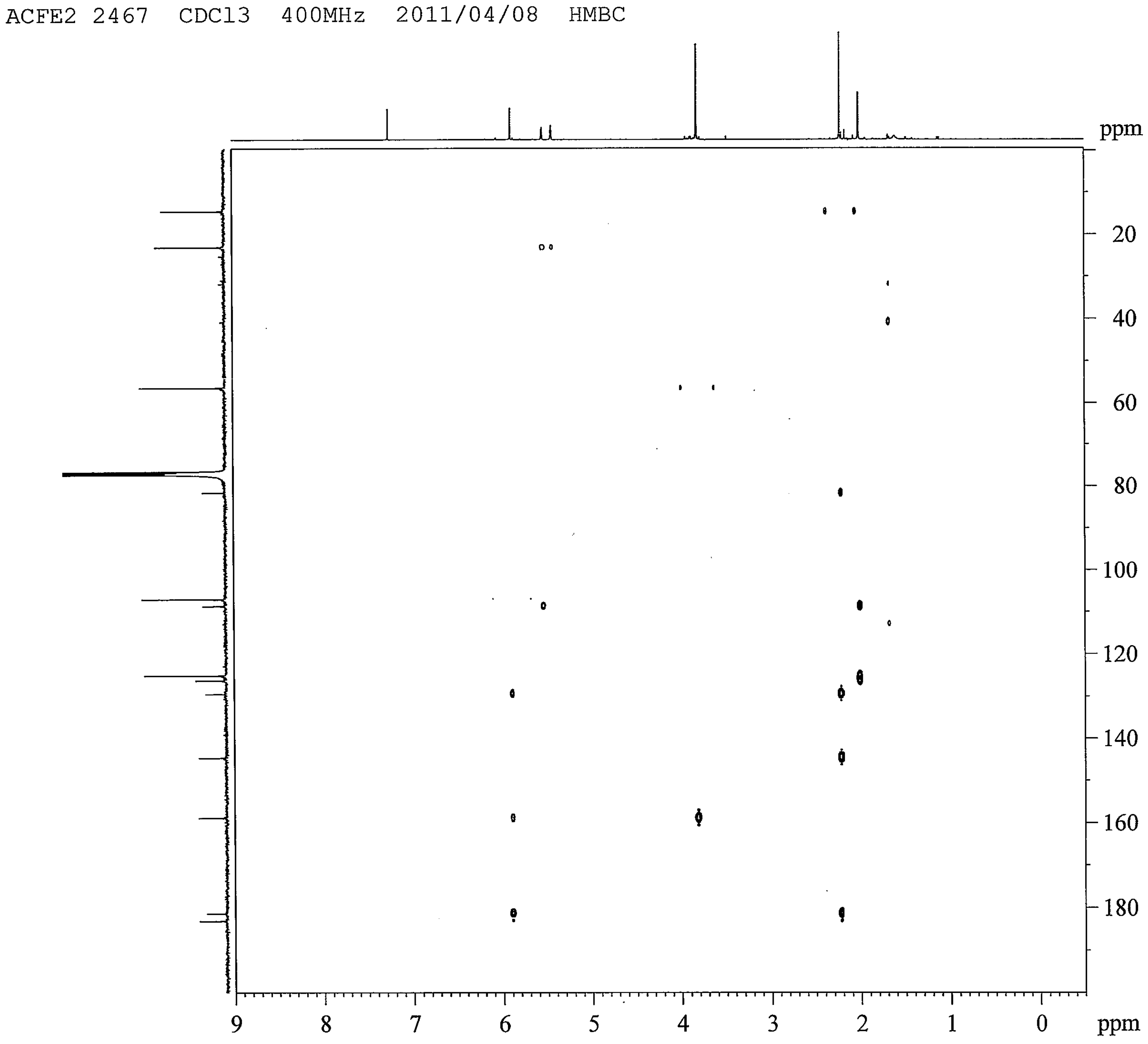

Fig. S27. NOESY spectrum of Benzocamphorin G (**3**)

ACFE2 2467 CDCl3 400MHz 2011/04/08 NOESY

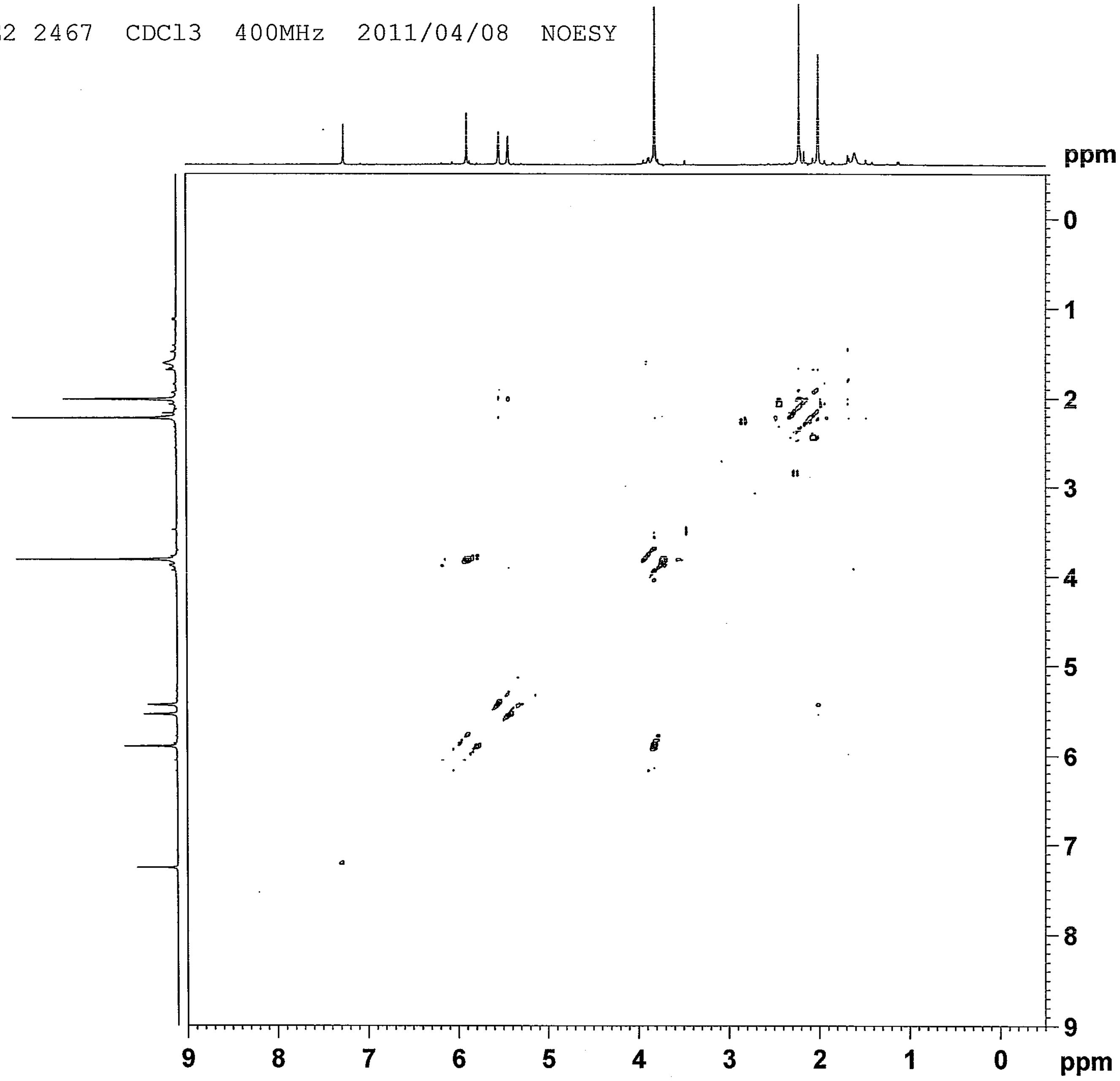

Fig. S28. ESI-MS spectrum of Benzocamphorin I (**4**)

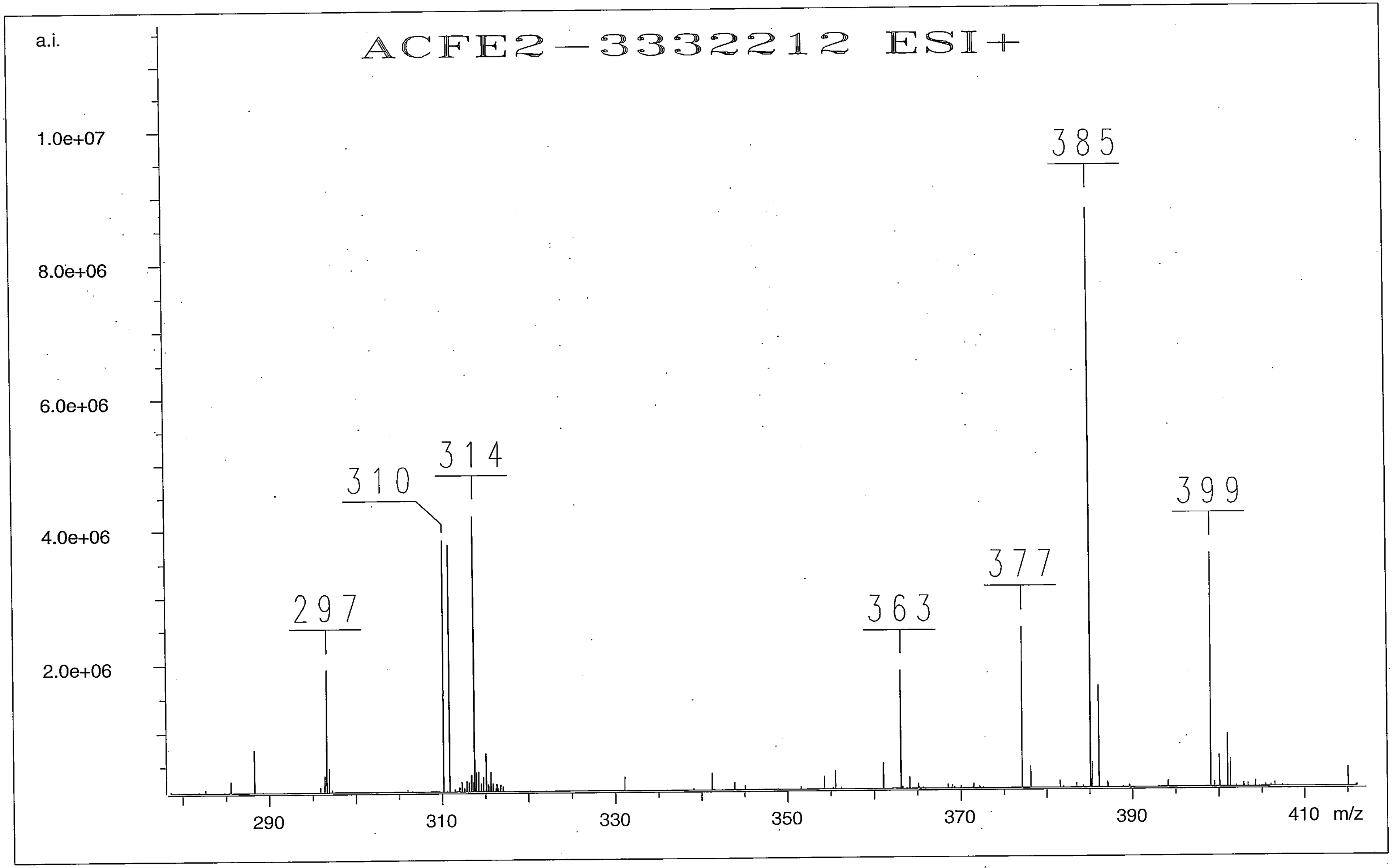

/d=/Data/yu/acfe23332212/1/pdata/1 Administrator Wed May 30 14:15:22 2012

Fig. S29. HRMS spectrum of Benzocamphorin I (**4**)

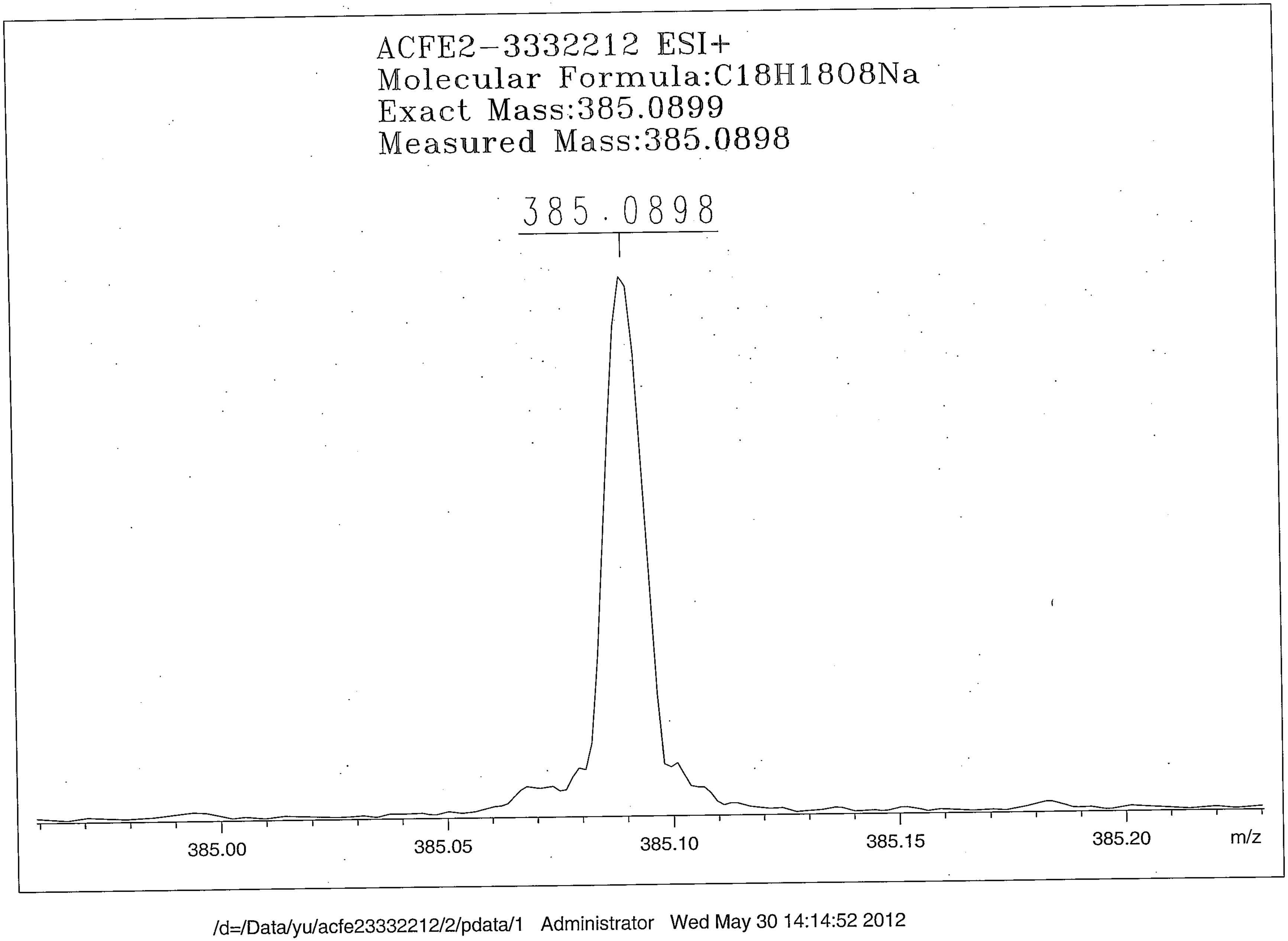

Fig. S30. IR spectrum of Benzocamphorin I (**4**)

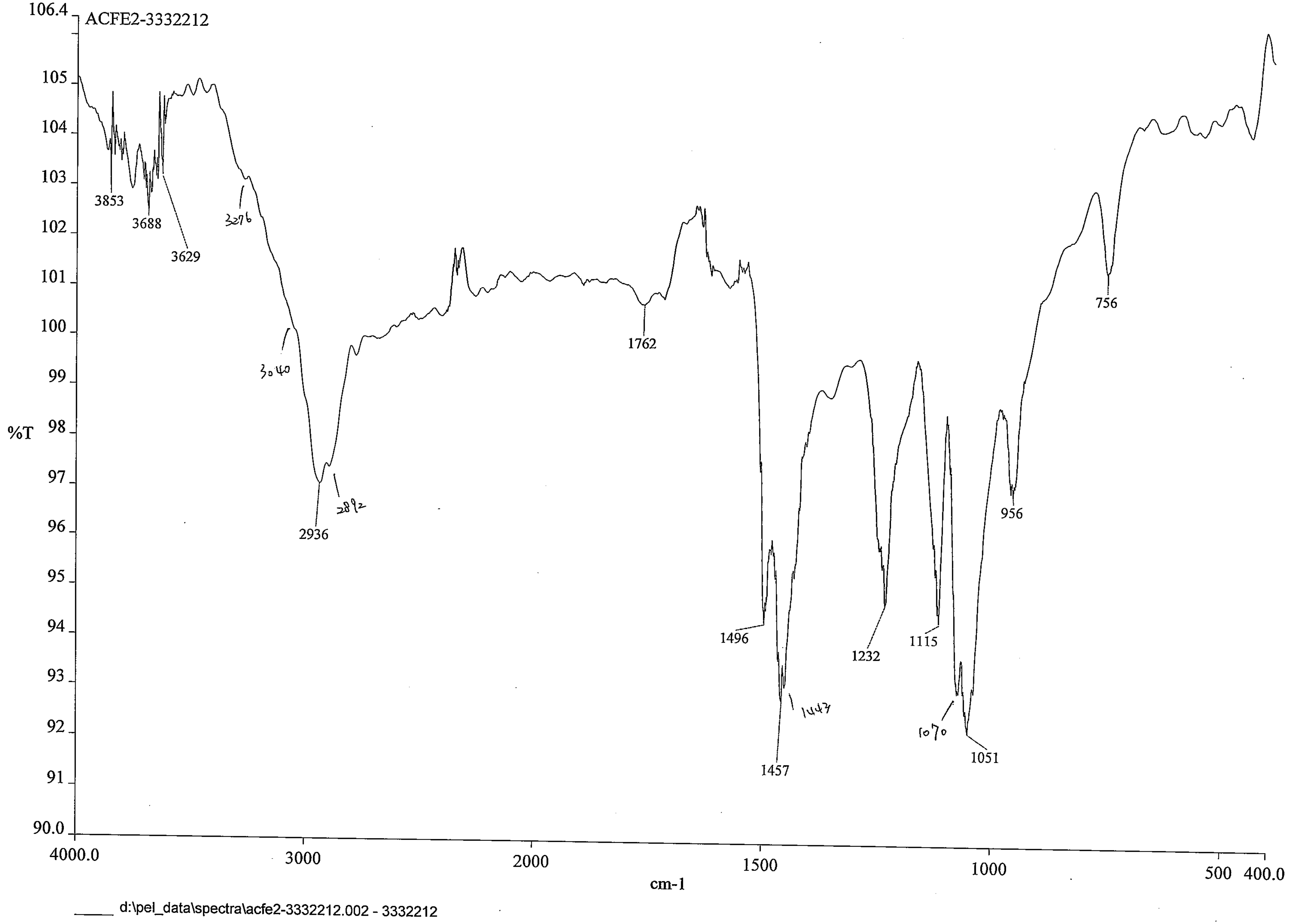

Fig. S31. ^{1}H NMR spectrum of Benzocamphorin I (**4**)

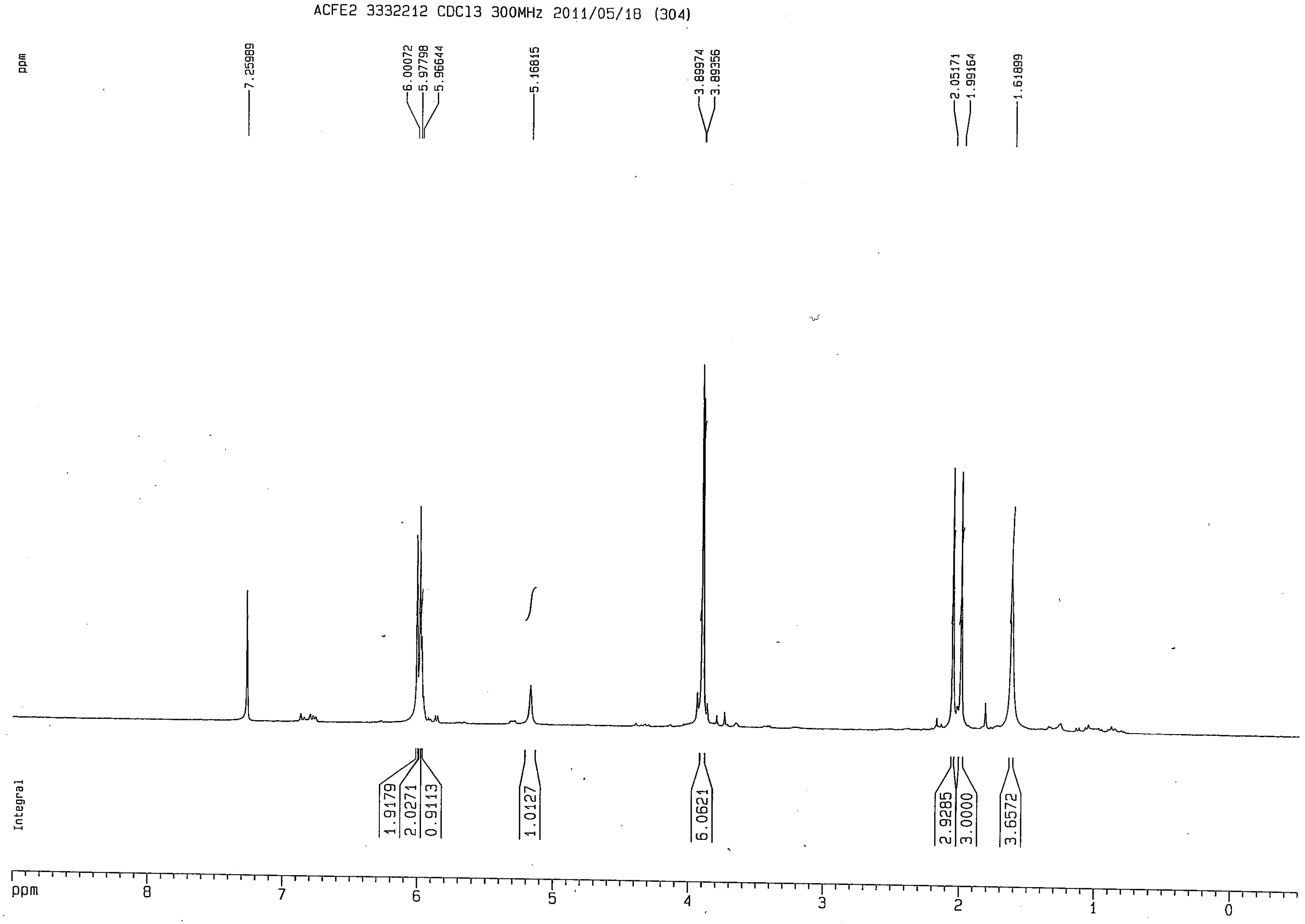

Fig. S32. ^{13}C and DEPT NMR spectrum of Benzocamphorin I (**4**)

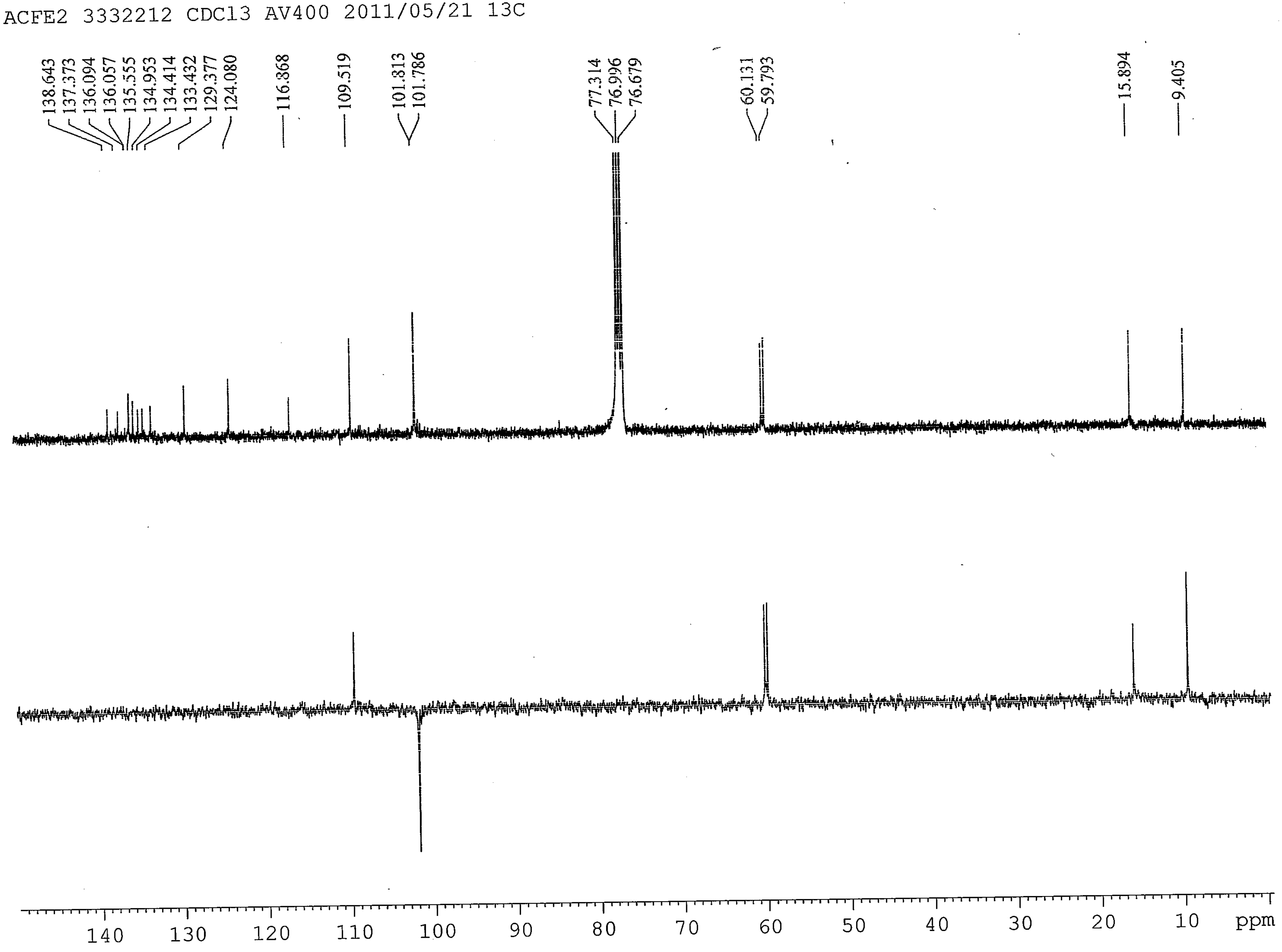

Fig. S33. COSY spectrum of Benzocamphorin I (**4**)

ACFE2 3332212 CDCl3 AV400 2011/05/21 COSY90

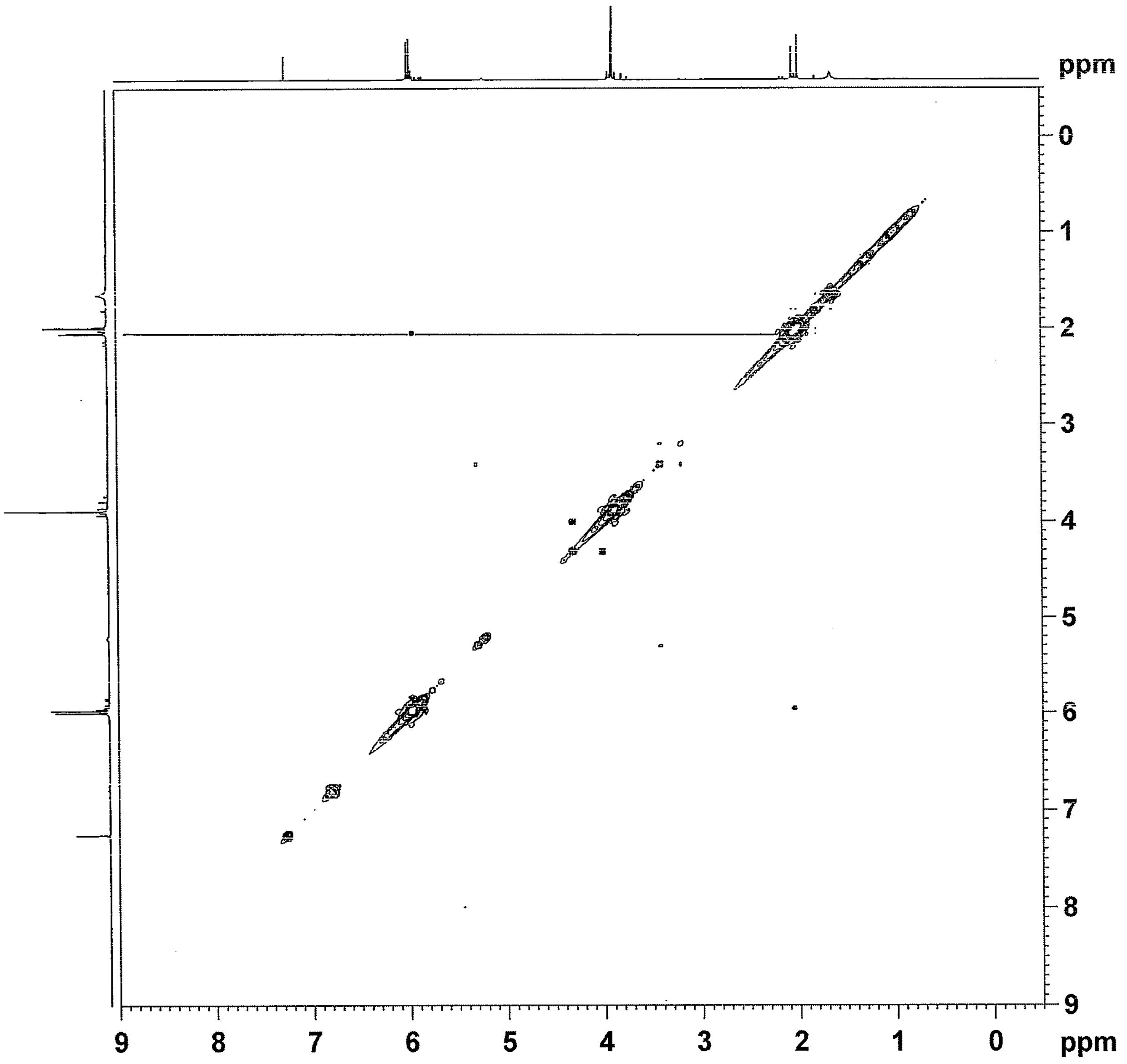

Fig. S34. HSQC spectrum of Benzocamphorin I (**4**)

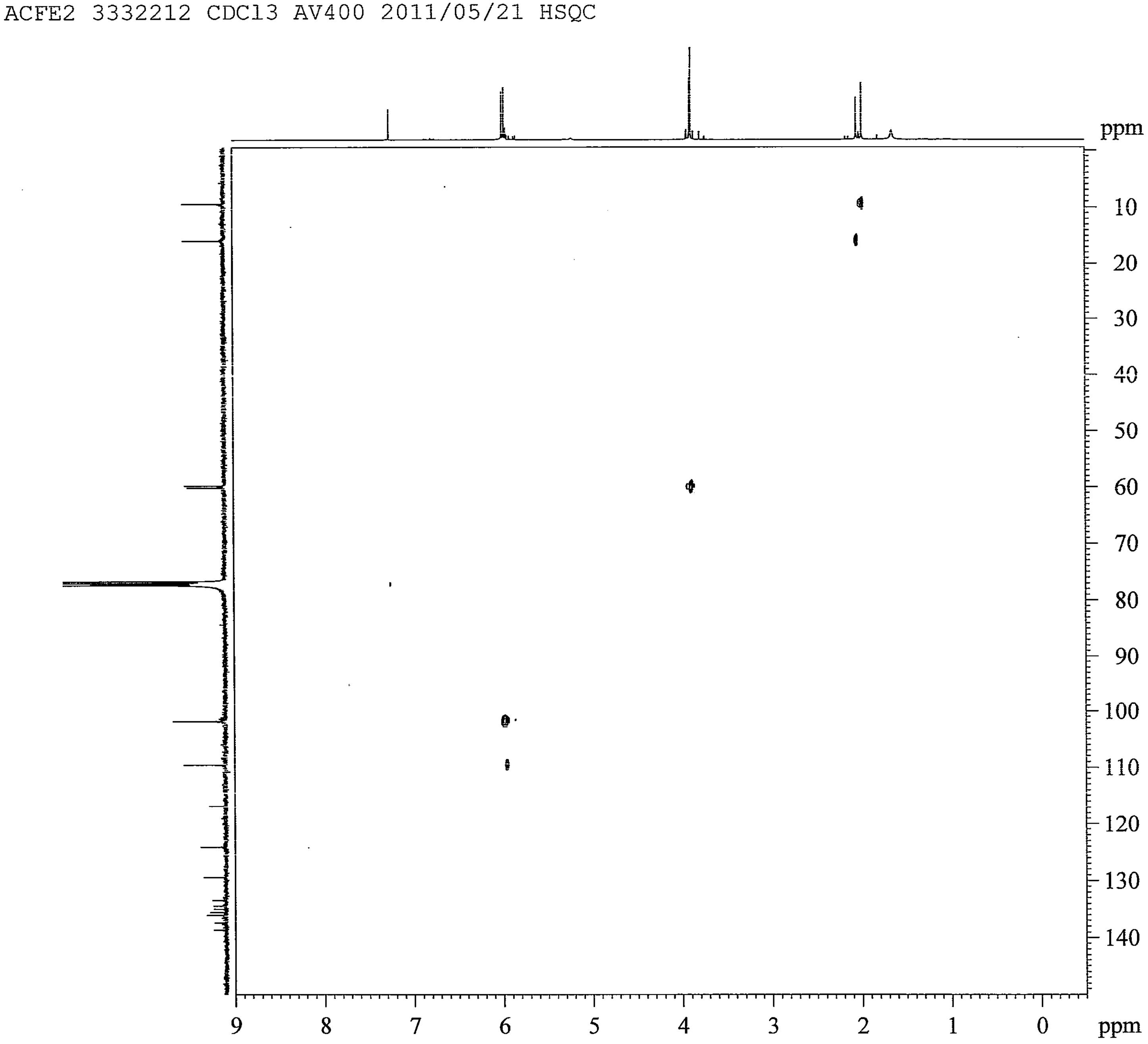

Fig. S35. HMBC spectrum of Benzocamphorin I (**4**)

ACFE2 3332212 CDCl3 AV400 2011/05/21 HMBC

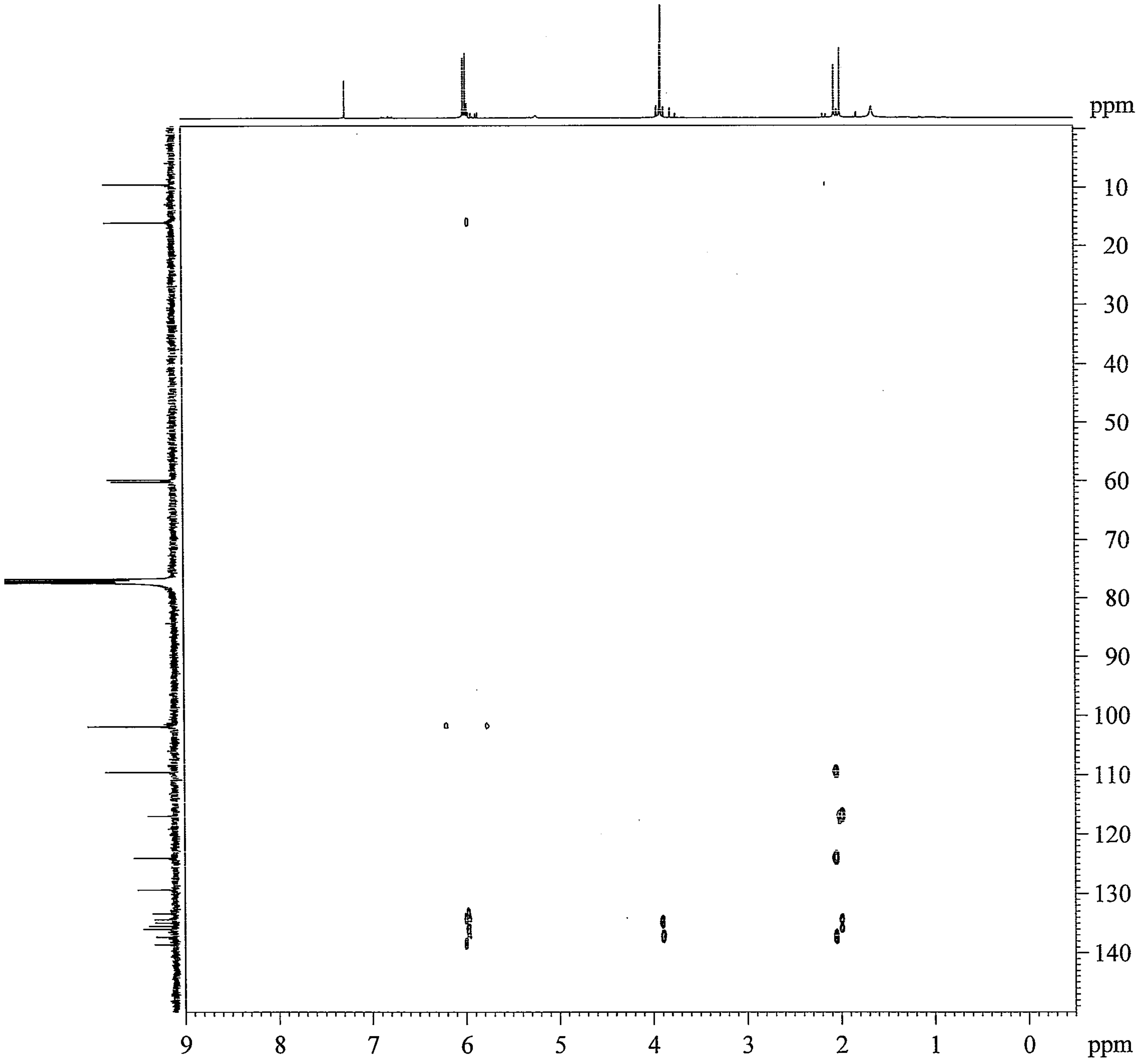

Fig. S36. NOESY spectrum of Benzocamphorin I (**4**)

ACFE2 3332212 CDCl3 AV400 2011/05/21 NOESY

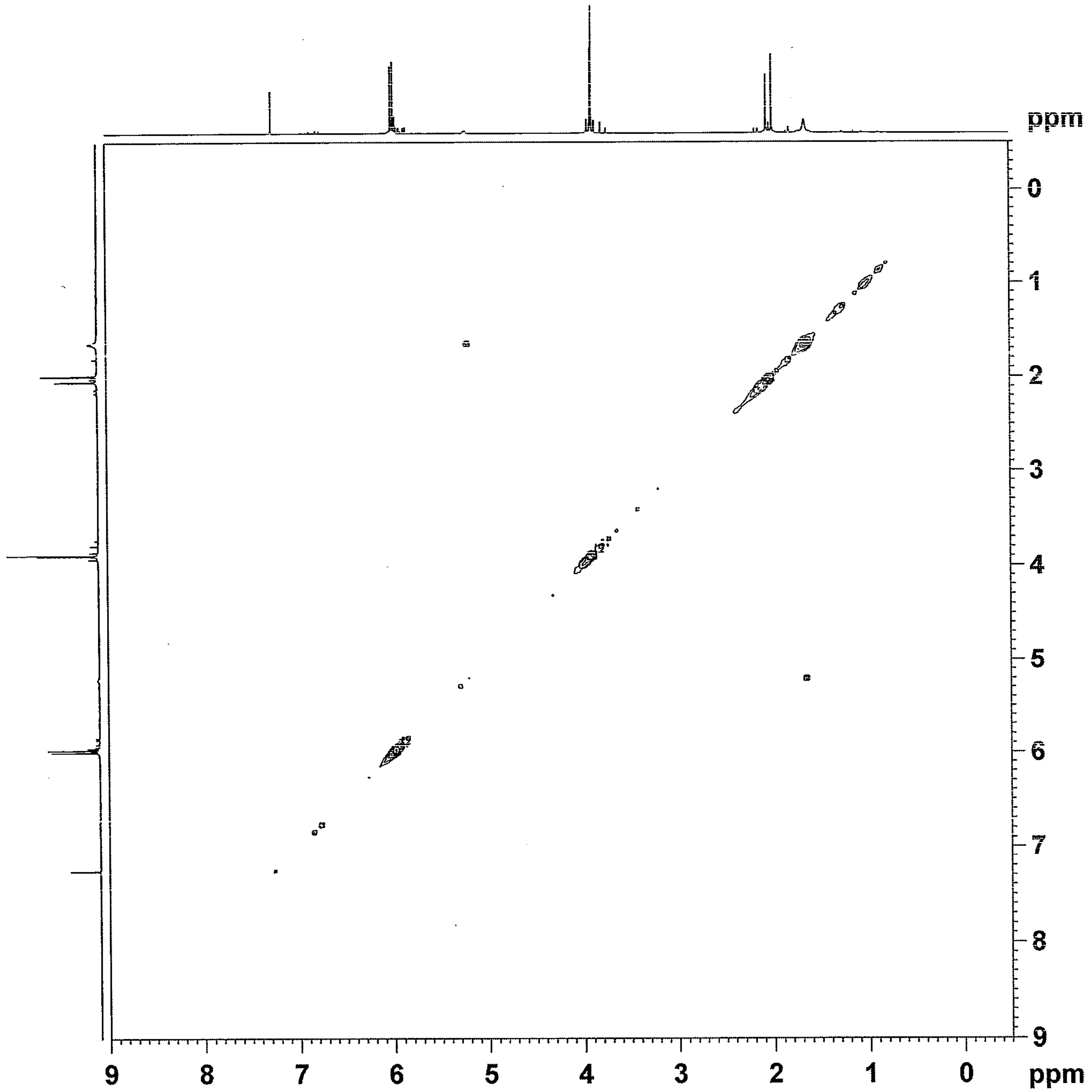

Supplement: Supplementary file 1 [file molecules-24-03730-s001.pdf]
